# Robust power and sample size calculations in quasi-likelihood models: methods and practice

**DOI:** 10.1186/s12874-026-02940-y

**Published:** 2026-07-21

**Authors:** Shijie Yuan, Amy Cochran, Paul Rathouz

**Affiliations:** 1 Department of Statistics and Data Sciences, The University of Texas at Austin, Austin, 78712 TX USA; 2 Department of Math, Department of Population Health Sciences, University of Wisconsin Madison, Madison, 53706 USA; 3 Department of Population Health, The University of Texas at Austin, Austin, 78712 TX USA

**Keywords:** 2SLiP, Effect size, Generalized linear models, P2R2, Quasi-likelihood models, Wald test, Score test

## Abstract

**Background:**

Accurate power and sample size calculations are essential in study planning, yet they are often difficult to carry out for quasi-likelihood (QL) models. Traditional power and sample size (PSS) approaches often rely on restrictive distributional assumptions, limiting their applicability when responses have non-standard distributions, variance functions are misspecified, or when covariates exhibit complex dependence structures.

**Methods:**

We examine whether two effect size measures—2 Standard Deviations in the Linear Predictor (2SLiP) and Pseudo-Partial $R^2$ (P2R2)—originally developed for Wald tests involving generalized linear models, are effective at power and sample size calculations in the QL framework. Through extensive simulations across diverse outcome types, link functions, and variance structures, we assess their performance under Wald tests and explore whether they remain useful for score tests. To illustrate practical utility, we apply these effect size measures to survey data on frontline health care workers to quantify the association between perceived personal protective equipment (PPE) adequacy and burnout risk during the COVID-19 pandemic, adjusting for covariates.

**Results:**

We show that the two generalized linear model (GLM)-based effect sizes are fundamentally moment-based objects, and therefore extend directly to QL models. Across all simulation settings, both measures remained accurate, with sample size and power estimates within 3% and 2% of the target, respectively. In the case study, the estimated effect sizes for perceived PPE adequacy (2SLiP = 0.096 and P2R2 = 0.020) correspond to small but meaningful associations with burnout risk after adjustment for demographic and occupational covariates; both yielded accurate recommendations for sample size.

**Conclusions:**

Both 2SLiP and P2R2 offer robust alternatives for power and sample size calculation in QL settings. By requiring minimal distributional assumptions, these measures enhance the flexibility and reliability of power and sample size calculations for realistic study designs commonly encountered in medical and public health research.

**Supplementary Information:**

The online version contains supplementary material available at https://doi.org/10.1186/s12874-026-02940-y.

## Introduction

Modern research increasingly relies on quasi-likelihood (QL) models to analyze outcomes ranging from binary events and counts to continuous measurements. Unlike generalized linear models (GLMs), which require full distributional specification within the exponential family, QL methods only posit a mean–variance relationship and a link function, allowing valid inference about the mean model under weaker assumptions [1]. This flexibility makes QL models especially attractive in applied research, where outcomes often deviate from canonical distributions, covariates exhibit complex correlation structures, and overdispersion is common. Yet, the same generality complicates study design: there remains a lack of generalized effect size measures that can accommodate arbitrary numbers of covariates as well as diverse outcome types, leaving power and sample size (PSS) estimation dependent on bespoke solutions for each new problem and setting.

For ease of discussion, we distinguish covariates into two categories: exposures and adjustors. Exposures refer to covariates of primary scientific interest whose associations or effects on the outcome are the main focus of inference, whereas adjustors are additional covariates included to control for confounding, account for nuisance variation, or improve estimation precision.

Over the past 40 years, a number of advances have been made within the framework of GLMs. Some, but not all, of these results might generalize to QL models, even when such extensions are not made explicit by the original authors. Several methods have specifically focused on the Wald test with a single exposure. Hauck and Donner [2] introduced the use of the Wald test in logistic regression for testing hypotheses on the coefficient of a single exposure, deriving the Fisher information and the conditional variance of the estimator. Whittemore [3] developed PSS approximations for logistic regression with rare outcomes, approximating the Fisher information through the moment-generating function of the covariates. Signorini [4] later extended this idea to the Poisson regression model, providing exact, albeit asymptotic, calculations without relying on approximation. Wilson and Gordon [5] proposed a general framework for PSS determination in GLMs that accounts for confounding variables by deriving approximate coefficient variances under both null and alternative hypotheses. Building on this, Shieh [6] refined Wald-based sample size methods by adjusting the variance of the tested coefficient according to the limiting values of nuisance parameters under the null hypothesis, extending earlier work on score and likelihood ratio tests [7, 8].

Shieh [9] and Lyles et al. [10] subsequently expanded Wald-based approaches to multiple exposures, with Lyles et al. [10] placing particular emphasis on nominal, count, and ordinal outcomes. Other contributions have addressed GLM-based PSS [11–16], though most remain limited to logistic or Poisson regression and specific covariate settings. Common software (e.g., SAS PROC POWER, G*Power, PASS) reflects these restrictions, focusing on logistic, Poisson, or analysis of variance (ANOVA)-style designs [17–19]. To address such limitations, Igeta et al. [20] proposed a QL-based framework for PSS calculation that explicitly incorporates possible misspecifications of the variance function in trials with over-dispersed count data. Beyond assumptions about the response distribution, traditional approaches also impose strong and often complex requirements on the joint distribution of exposures and adjustors, as well as on the exact outcome– exposure relationships. These demands limit their practical use, especially with multiple exposures or complex study designs [12, 14], such as when covariates arise as random processes in observational studies. As a result, researchers are frequently forced to adopt unrealistic assumptions or oversimplified models, reducing both the reliability of findings and the efficiency of resource use.

Cochran et al. [21] addressed these limitations by proposing two effect size measures for PSS calculations applicable to any GLM. For ease of reference, we refer to these as 2 Standard deviations in Linear Predictor (2SLiP), which captures the additional variance explained by exposures in the linear predictor scale beyond adjustors, and Pseudo-Partial $R^2$ (P2R2), which generalizes partial $R^2$ to GLMs by quantifying the proportion of outcome variance on a standard deviation scale explained by exposures beyond adjustors. These measures were designed to benefit from the same properties that make the framework described by Gatsonis and Sampson [22] useful for study planning in linear regression, namely, interpretability, support for joint testing of exposures, adjustment for arbitrary covariates, and reliance only on first and second moment information. Because the development of these effects sizes is restricted to GLMs, this restriction implicitly ties the proposed effect size measures to fully specified likelihoods. By exploiting the fact that 2SLiP and P2R2 depend only on first- and second-moment information, we extend the framework to QL models, broadening its applicability while preserving interpretability and accessibility.

This article extends prior methodological work in four ways: First, we launch from a QL framework, a semi-parametric generalization of GLMs that requires only specification of the linear predictor, link, and variance function, without a full probability model [23]. Second, we examine whether the proposed PSS calculations remain useful when applied to score tests, moving beyond the Wald test focus of earlier work. Third, we evaluate the performance of 2SLiP and P2R2 through simulations across diverse QL settings, including overdispersed Poisson, dispersed gamma, and non-canonical links, to assess accuracy and robustness under realistic data structures. Fourth, we present a case study using survey data from Cahill et al. [24] to demonstrate how these measures can be applied in practice, linking effect size estimates to sample size requirements in studies of perceived personal protective equipment (PPE) adequacy and mental health.

The remainder of this article is organized as follows. Methods section reviews the proposed effect size measures, 2SLiP and P2R2, and extends the methodological framework to QL models and score tests. Simulations section reports simulation studies evaluating their performance across settings. Case study section presents a case study illustrating their practical application. Discussion section discusses the results, practical implications, and directions for future research.

## Methods

### Preliminaries

Consider an outcome * Y * for a single unit together with its exposures of primary interest $\boldsymbol{X}$ and adjustors $\boldsymbol{Z}$. The vector of exposures $\boldsymbol{X}$ is * p *-dimensional, while $\boldsymbol{Z}$ is * r *-dimensional capturing the adjustment covariates, including an intercept term.

Under QL theory [25], the user specifies the mean and variance of *Y* conditional on $\boldsymbol{X}$ and $\boldsymbol{Z}$ as

$$\begin{aligned}&\mathbb {E}[Y \mid \boldsymbol{X}, \boldsymbol{Z}] = \mu (\boldsymbol{X},\boldsymbol{Z}) \quad \text {and}\\&\text {Var}(Y \mid \boldsymbol{X}, \boldsymbol{Z}) = \sigma ^2 v(\mu (\boldsymbol{X},\boldsymbol{Z})), \end{aligned}$$

where $\sigma ^2$ is a dispersion parameter and *v(· )* is a known variance function. We refer to $\mu (\boldsymbol{X},\boldsymbol{Z})$ simply by *μ * and $v(\mu (\boldsymbol{X},\boldsymbol{Z}))$ by *v* when unambiguous. The mean *μ * is related to the exposures and adjustors through a known link function $g(\mu ) = \boldsymbol{\lambda }' \boldsymbol{Z} + \boldsymbol{\beta }' \boldsymbol{X} \equiv \eta$, where *η * is the linear predictor, and $\boldsymbol{\lambda }$ and $\boldsymbol{\beta }$ are the parameter vectors.

Parameters $\boldsymbol{\lambda }$ and $\boldsymbol{\beta }$ are estimated by solving the quasi-score equations. These equations are recovered from the quasi-score function, given by

1 $$\begin{aligned} \boldsymbol{U}(\boldsymbol{\lambda }, \boldsymbol{\beta }) = \frac{Y - \mu }{\sigma ^2 v(\mu )} \left( \frac{d\mu }{d\eta } \right) \left[ \begin{array}{c} \boldsymbol{Z} \\ \boldsymbol{X} \end{array}\right] . \end{aligned}$$

In practice, parameter estimates are obtained by setting the sample mean of the quasi-score function to zero. In the population, this corresponds to solving $\mathbb {E}[\boldsymbol{U}(\boldsymbol{\lambda }, \boldsymbol{\beta })] = 0$ with expectation taken over the true joint distribution of $(Y,\boldsymbol{X},\boldsymbol{Z})$. Solutions to this population equation, denoted by $(\boldsymbol{\lambda }^*, \boldsymbol{\beta }^*)$, define the population QL parameter values.

Directly solving the population quasi-score equations is generally infeasible due to the nonlinearity of the link function. However, an equivalent characterization is provided by iteratively weighted least squares (IRLS) [23]. Specifically, by taking a first-order Taylor expansion of the outcome *Y* transformed to the linear predictor scale and expanded about its mean, we obtain a working linearized outcome for each observation: $Y_l \equiv \eta + \frac{d\eta }{d\mu }(Y-\mu ).$ The quasi-score equations can then be replaced by normal equations of a weighted least squares problem, in which $Y_l$ is repeatedly regressed onto *η * with weights for a single observation given by

2 $$\begin{aligned} w \equiv \frac{1}{\sigma ^2 v(\mu )} \left( \frac{d\mu }{d\eta }\right) ^2. \end{aligned}$$

This can be equivalently expressed with the first-order conditions for minimizing a weighted mean squared error:

3 $$\begin{aligned} (\boldsymbol{\lambda }^*,\boldsymbol{\beta }^*) = \underset{\boldsymbol{\lambda },\boldsymbol{\beta }}{\arg \,\min } \mathbb {E}\left[ w \left\{ Y_l - \big (\boldsymbol{\lambda }' \boldsymbol{Z} + \boldsymbol{\beta }' \boldsymbol{X}\big ) \right\} ^2 \right] . \end{aligned}$$

### Measures of effect size

The key observation of the present paper is that the weighted mean squared error in Equation (3) has exactly the same form as in the GLM setting: it depends only on the conditional mean of *Y* given $(\boldsymbol{X},\boldsymbol{Z})$, as specified by the link function and linear predictor, and on the conditional variance of *Y*. In GLMs, this variance is determined by the assumed distributional family, whereas under QL it is specified directly as $\text {Var}(Y\mid \boldsymbol{X},\boldsymbol{Z})=\sigma ^2 v(\mu )$, without otherwise specifying the distribution of *Y*. Consequently, any construction that depends only on the weighted least squares representation—and not on a fully specified likelihood—generalizes from the GLM to the QL setting.

In particular, the effect size measures introduced for GLMs by Cochran et al. [21] are of this form, depending only on the weighted least squares representation. We therefore adopt the same constructions here, with expectations and weights taken under the QL specification. To formalize these measures in the current setting, we first introduce the reduced model that includes only the adjustors $\boldsymbol{Z}$. Let

4 $$\begin{aligned} \boldsymbol{\lambda }^*_0 = \underset{\boldsymbol{\lambda }}{\arg \,\min } \mathbb {E}\!\left[ w \left\{ Y_l - \boldsymbol{\lambda }' \boldsymbol{Z} \right\} ^2 \right] , \end{aligned}$$

where *w* and $Y_l$ are evaluated as in (3) at the true QL parameter values $(\boldsymbol{\lambda }^*,\boldsymbol{\beta }^*)$. The reduced-model linear predictor and conditional mean are then given by

$$\begin{aligned} \eta _z = {\boldsymbol{\lambda }^*_0}' \boldsymbol{Z}, \qquad \mu _z = g^{-1}(\eta _z). \end{aligned}$$

Using these quantities, we define two effect size measures. The first is a 2 Standard deviation contrast on the Linear Predictor scale (2SLiP),

5 $$\begin{aligned} \phi _{x\mid z} \equiv 2\,\sqrt{\text {Var}(\eta _{x|z})}, \end{aligned}$$

where $\eta _{x|z} \equiv \eta - \eta _z$. With $\eta _{x|z}$ representing the difference between the full linear predictor *η * and the reduced-model linear predictor $\eta _z$ that includes only $\boldsymbol{Z}$, 2SLiP quantifies the variability in the linear predictor attributable to the exposures $\boldsymbol{X}$ beyond the adjustors $\boldsymbol{Z}$. The second measure is a Pseudo-Partial *R*-squared (P2R2),

6 $$\begin{aligned} R^{2}_{x\mid z} \equiv \frac{\mathbb {E}\left[ (Y - \mu _z)^2 / \{\sigma ^2 v(\mu )\}\right] - \mathbb {E}\!\left[ (Y - \mu )^2 / \{\sigma ^2 v(\mu )\}\right] }{\mathbb {E}\left[ (Y - \mu _z)^2 / \{\sigma ^2 v(\mu )\}\right] }. \end{aligned}$$

It measures the proportion of outcome variability on a standard deviation scale explained by the full model mean *μ * beyond the reduced-model mean $\mu _z$ that includes only $\boldsymbol{Z}$. In the following section, we outline how these effect size measures are employed to obtain approximate power calculations, and we provide theoretical justification for these approximations in Supplementary Section S.2.

Because both effect size measures depend only on the first two conditional moments of Y given $(\boldsymbol{X},\boldsymbol{Z})$, their interpretation can be anchored in the GLM setting and carried over. For example, in Poisson regression with a log link, 2SLiP corresponds to the log rate ratio for *Y* contrasting two units that differ by two standard deviations in $\eta _{x|z}$; for a balanced binary exposure $\boldsymbol{X}$ with no adjustors, this is simply the contrast between $\boldsymbol{X}=1$ and $\boldsymbol{X}=0$. In linear regression with unit error variance, 2SLiP reduces to Cohen’s d [26], and P2R2 is exactly the partial $R^2$. The only distinction in the QL setting is that the conditional variance, and hence the weights, may include a dispersion parameter $\sigma ^2$, which affects the scaling of variability but not the interpretation of the effect size. In an overdispersed Poisson quasi-likelihood model, for instance, 2SLiP retains the interpretation of a log rate ratio over a two–standard deviation contrast in $\eta _{x|z}$.

### Power calculations

The effect size measures introduced are intended to support power and sample size calculations across a broad class of models, including those not fully specified by a parametric family. In GLMs, these measures can be used directly in Wald test power calculations. We ask whether this approach is also appropriate for QL models, considering both Wald and score tests evaluating the null hypothesis that the exposures of interest have no effect, i.e. $H_0:\boldsymbol{\beta }^* = \boldsymbol{0}$. We review the relevant tests and describe how the effect sizes are applied.

#### Wald test.

In QL models with *n* independent samples, the Wald statistic is constructed from the QL estimators of the model parameters and their estimated covariance, denoted by $(\widehat{\boldsymbol{\lambda }}, \widehat{\boldsymbol{\beta }})$ and $\boldsymbol{i}_n^{-1}(\widehat{\boldsymbol{\lambda }}, \widehat{\boldsymbol{\beta }})$, respectively. The matrix $\boldsymbol{i}_n(\boldsymbol{\lambda }, \boldsymbol{\beta })$ is the expected Fisher information matrix in QL theory and can be computed from the quasi-score function $\boldsymbol{U}_i(\lambda ,\beta )$ evaluated at i.i.d. observations $(Y_i, \boldsymbol{X}_i, \boldsymbol{Z}_i)$ for *i=1* to *i=n*. Compactly, we can write this matrix as

7 $$\begin{aligned} \boldsymbol{i}_n(\boldsymbol{\lambda },\boldsymbol{\beta })&= \sum _{i=1}^n \mathbb {E}\left[ - \frac{\partial \boldsymbol{U}_i(\boldsymbol{\lambda }, \boldsymbol{\beta })}{\partial (\boldsymbol{\lambda }, \boldsymbol{\beta })} \bigg | \boldsymbol{X}_i, \boldsymbol{Z}_i\right] \nonumber \\ &= \sum _{i = 1}^n w_i \left[ \begin{array}{cc} \boldsymbol{Z}_i\boldsymbol{Z}_i' & \boldsymbol{Z}_i\boldsymbol{X}_i'\\ \boldsymbol{X}_i\boldsymbol{Z}_i' & \boldsymbol{X}_i\boldsymbol{X}_i' \end{array}\right] , \end{aligned}$$

whose dependence on $\boldsymbol{\lambda }$ and $\boldsymbol{\beta }$ enters only through $w_i$, representing the weight term evaluated at the observation $(Y_i, \boldsymbol{X}_i, \boldsymbol{Z}_i)$. That is, $w_i = (\frac{d\mu }{d\eta } |_{\eta = \eta _i})^2 / \left( \sigma ^2 v(\mu _i) \right)$ with $\eta _i = \boldsymbol{\lambda }'\boldsymbol{Z}_i + \boldsymbol{\beta }'\boldsymbol{X}_i$ and $\mu _i = g^{-1}(\eta _i)$. Taking $\boldsymbol{i}_{n,\boldsymbol{\beta }|\boldsymbol{\lambda }}^{-1}(\boldsymbol{\lambda },\boldsymbol{\beta })$ to be the lower right *p × p* block of $\boldsymbol{i}_{n}^{-1}(\boldsymbol{\lambda },\boldsymbol{\beta })$, the Wald test statistic becomes $\widehat{W} = \widehat{\boldsymbol{\beta }}' \boldsymbol{i}_{n,\boldsymbol{\beta }|\boldsymbol{\lambda }}(\widehat{\boldsymbol{\lambda }}, \widehat{\boldsymbol{\beta }}) \widehat{\boldsymbol{\beta }}$.

Asymptotically, the Wald statistic $\widehat{W}$ follows a non-central chi-squared distribution $\chi ^2_p(\Delta )$ with *p* degrees of freedom and non-centrality parameter $\Delta \equiv n f^{2}$ [25]. A formal derivation of the asymptotic distribution of the Wald statistic in the quasi-likelihood setting is provided in Supplementary Section S.1. The effect size measure $f^{2}$ is defined as

8 $$\begin{aligned} f^{2} \equiv {\boldsymbol{\beta }^*}' \boldsymbol{\mathcal {I}}_{\boldsymbol{\beta } | \boldsymbol{\lambda }} \boldsymbol{\beta }^*, \end{aligned}$$

where

9 $$\begin{aligned} \boldsymbol{\mathcal {I}}_{\boldsymbol{\beta } | \boldsymbol{\lambda }} \equiv \mathbb {E}\left[ w \boldsymbol{X} \boldsymbol{X}' \right] - \mathbb {E}\left[ w \boldsymbol{X} \boldsymbol{Z}' \right] \mathbb {E}\left[ w \boldsymbol{Z} \boldsymbol{Z}' \right] ^{-1} \mathbb {E}\left[ w \boldsymbol{Z} \boldsymbol{X}' \right] , \end{aligned}$$

obtained as the Schur complement of the block $\mathbb {E}[w \boldsymbol{Z} \boldsymbol{Z}']$ in the marginal Fisher information matrix:

10 $$\begin{aligned} \boldsymbol{\mathcal {I}} = \left[ \begin{array}{cc} \mathbb {E}[w \boldsymbol{Z} \boldsymbol{Z}'] & \mathbb {E}[w \boldsymbol{Z} \boldsymbol{X}'] \\ \mathbb {E}[w \boldsymbol{X} \boldsymbol{Z}'] & \mathbb {E}[w \boldsymbol{X} \boldsymbol{X}'] \end{array}\right] . \end{aligned}$$

Note $\boldsymbol{\mathcal {I}}_{\boldsymbol{\beta } | \boldsymbol{\lambda }}^{-1}$ corresponds to the lower right *p × p* block of $\boldsymbol{\mathcal {I}}^{-1}$.

Once you have the effect size $f^2$, calculating the asymptotic power of the Wald test is straightforward. Since the Wald statistic follows a central chi-squared distribution under the null hypothesis, you would reject at significance level *α * if $\widehat{W}$ exceeds the *(1-α )*-quantile of $\chi ^2_p(0)$. Under the alternative hypothesis, the asymptotic power is then simply the probability that a non-central chi-squared distribution with non-centrality parameter $\Delta = n f^2$ exceeds the same critical value used under the null. Formally, the asymptotic power is given by

11 $$\begin{aligned} q = 1 - F_{\chi ^2_p(\Delta )} \left( F^{-1}_{\chi ^2_p(0)}(1 - \alpha ) \right) . \end{aligned}$$

is the cumulative distribution function of the non-central chi-squared distribution. In other words, once you have $f^2$ and the sample size *n*, you can immediately compute asymptotic power for any chosen significance threshold.

#### Approximate power.

Because $f^2$ depends on population quantities that are typically unknown, we approximate it using the effect size measures 2SLiP (5) and P2R2 (6), and substitute these approximations into the power expression above. Formal error analyses are available in the GLM setting [21], and because these arguments depend only on first and second moments, they apply equally to the present QL framework. Below, we present the intuition behind why these approximations work in this broader context.

Both measures are grounded in the identity $f^2 = \mathbb {E}[ w \eta _{x|z}^2 ]$, with theoretical justification provided in Supplementary Section S.2. For 2SLiP, expanding this expectation using basic moment identities yields

12 $$\begin{aligned} f^{2} = \mathbb {E}[w] \left( \mathbb {E}[\eta _{x|z}]^2 + \phi _{x|z}^2 / 4\right) + \text {cov}\left[ w, \eta _{x|z}^2\right] . \end{aligned}$$

This decomposition clarifies when a simple approximation is reasonable. In settings where the weights exhibit little variation relative to $\phi _{x|z}$, the mean contribution $\mathbb {E}[\eta _{x|z}]$ is small, the covariance term $\text {cov}[w,\eta _{x|z}^2]$ is negligible, and $\mathbb {E}[w]$ is well approximated by a constant $w_{1\!\!1}$. Under these conditions, the effect size $f^2$ is well approximated by

$$\begin{aligned} f^2 \approx f^2_{\phi } \equiv w_{1\!\!1} \phi _{x|z}^2 / 4. \end{aligned}$$

This approximation is exact when weights are constant, as in linear regression. In practice, we set $w_{1\!\!1}$ to the weight evaluated at $\mu = \mathbb {E}[Y]$, since the marginal mean can often be specified.

For P2R2, we proceed slightly differently. A first-order Taylor expansion of the conditional mean around $\eta _z$ yields

13 $$\begin{aligned} \frac{d\mu }{d\eta } (\eta -\eta _z) = \frac{d\mu }{d\eta } \eta _{x|z} \approx (\mu - \mu _z). \end{aligned}$$

Substituting this approximation into the identity $f^2 = \mathbb {E}[w\,\eta _{x|z}^2]$ gives

$$\begin{aligned} f^2 \approx f^2_R \equiv \mathbb {E}\left[ \frac{ (\mu - \mu _z)^2}{\sigma ^2 v(\mu )} \right] = \frac{R^{2}_{x\mid z}}{1-R^{2}_{x\mid z}}. \end{aligned}$$

This approximation is exact if the link function *g(· )* is linear, as with the identity link. For nonlinear mean functions, the approximation is accurate when $\eta _{x|z}$ is close to zero, so that the first-order Taylor expansion about $\eta _z$ provides a good local approximation.

#### Score Test.

The preceding develop uses 2SLiP and P2R2 to approximate the non-centrality parameter governing the asymptotic power of the Wald test. While this provides a principled framework, it is ultimately an approximation rooted in large-sample theory, whereas power in practice is determined by finite-sample behavior. This motivates examining whether the same effect-size–based approximation strategy extends is a good heuristic for other testing procedures. To investigate this, we review the score test in the QL setting.

Let $\boldsymbol{U}_n(\boldsymbol{\lambda },\boldsymbol{\beta })$ denote the function obtained by summing the individual quasi-scores $\boldsymbol{U}_i(\boldsymbol{\lambda },\boldsymbol{\beta })$ over *n* i.i.d. observations $(Y_i,\boldsymbol{X}_i,\boldsymbol{Z}_i)$, and let $\boldsymbol{U}_{n\boldsymbol{\lambda }}(\boldsymbol{\lambda },\boldsymbol{\beta })$ represents the part of the vector $\boldsymbol{U}_n(\boldsymbol{\lambda },\boldsymbol{\beta })$ corresponding to $\boldsymbol{\lambda }$. The score test is based on the restricted estimator $\widehat{\boldsymbol{\lambda }}^{(0)}$, defined as the solution to $\boldsymbol{U}_{n\boldsymbol{\lambda }}(\boldsymbol{\lambda },\boldsymbol{0})=\boldsymbol{0}$. The resulting score statistic is:

14 $$\begin{aligned} \widehat{S} = \boldsymbol{U}_n\left( \widehat{\boldsymbol{\lambda }}^{(0)}, \boldsymbol{0}\right) '\boldsymbol{i}_n^{-1}\left( \widehat{\boldsymbol{\lambda }}^{(0)}, \boldsymbol{0}\right) \boldsymbol{U}_n\left( \widehat{\boldsymbol{\lambda }}^{(0)}, \boldsymbol{0}\right) . \end{aligned}$$

Under the null hypothesis $\boldsymbol{\beta }^*=\boldsymbol{0}$, $\widehat{S}$ converges in distribution to a central chi-squared distribution with *p* degrees of freedom. Under the alternative $\boldsymbol{\beta }^*\ne \boldsymbol{0}$, however, its limiting distribution is no longer chi-squared, which complicates power calculations.

This complication arises because $\widehat{\boldsymbol{\lambda }}^{(0)}$ is generally not a consistent estimator of $\boldsymbol{\lambda }^*$ when $\boldsymbol{\beta }^*\ne \boldsymbol{0}$. Instead, it converges to the solution $\boldsymbol{\lambda }^{(0)}$ to the population equation $\mathbb {E}[\boldsymbol{U}_{\boldsymbol{\lambda }}(\boldsymbol{\lambda },\boldsymbol{0})]=\boldsymbol{0}$ where $\boldsymbol{U}_{\boldsymbol{\lambda }}(\boldsymbol{\lambda },\boldsymbol{0})$ is the part of the quasi-score $\boldsymbol{U}(\boldsymbol{\lambda },\boldsymbol{\beta })$ corresponding to $\boldsymbol{\lambda }$. As a result, determining the asymptotic behavior of the score statistic under the alternative requires characterizing both $\boldsymbol{\lambda }^{(0)}$ and the corresponding mean and covariance of $\boldsymbol{U}_n(\widehat{\boldsymbol{\lambda }}^{(0)},\boldsymbol{0})$. While these quantities simplify under the null, they do not admit comparably simple expressions under the alternative, making exact power calculations difficult.

Given this difficulty, we adopt a simple heuristic to help benchmark how our power approximations based on 2SLiP and P2R2 perform for score tests in finite samples. Specifically, we define an approximate effect size for the score test by replacing the random components of the score statistic with their population counterparts:

15 $$\begin{aligned} f_s^2 \equiv \mathbb {E}\left[ \boldsymbol{U}(\boldsymbol{\lambda }^{(0)}, \boldsymbol{0})'\right] \mathbb {E}\left[ \boldsymbol{i}^{-1}(\boldsymbol{\lambda }^{(0)}, \boldsymbol{0})\right] \mathbb {E}\left[ \boldsymbol{U}(\boldsymbol{\lambda }^{(0)}, \boldsymbol{0})\right] . \end{aligned}$$

Here, $\mathbb {E}[\boldsymbol{i}^{-1}(\boldsymbol{\lambda }^{(0)}, \boldsymbol{0})]$ represent the marginal Fisher information, obtained by substituting $\boldsymbol{\lambda } = \boldsymbol{\lambda }^{(0)}$ and $\boldsymbol{\beta } = \boldsymbol{0}$ into (10). As with $f^2$, $f_\phi ^2$, and $f_R^2$, we use $f_s^2$ by substituting $n f_s^2$ for the noncentrality parameter in the corresponding asymptotic power calculation. This approximation is motivated by behavior near the null and is intended solely as a benchmark rather than an exact characterization.

## Simulations

The simulation study evaluates the robustness of 2SLiP and P2R2 for Wald and score tests under GLM and QL settings, assessing whether these measures continue to provide stable and interpretable PSS estimates when standard likelihood assumptions are relaxed. A key focus of this study is on cases where the outcome variable is either a count or a positive continuous quantity. For count outcomes, we examine different link functions (log and identity) and variance structures: variance equal to the mean, proportional to the mean, or proportional to the squared mean. For positive continuous outcomes, we evaluate cases where the variance is proportional to either the mean or the squared mean under a log-link function.

We chose these variance structures to cover both canonical GLM settings and common quasi-likelihood departures, thereby evaluating performance across a range of mean–variance relationships and weight functions. For count outcomes, the Poisson variance structure (var = mean) provides a natural baseline, but many real-world count data are overdispersed, with variability exceeding that implied by the Poisson model. Such behavior is common in event counts, healthcare utilization outcomes, and clustered or heterogeneous populations [27–29]. For example, counts of Chronic Obstructive Pulmonary Disease (COPD) exacerbations are often modeled using negative binomial (NB) regression because substantial between-patient heterogeneity induces overdispersion and variance growth beyond the Poisson assumption [30]. For positive continuous outcomes, the variance structure var *∝ * mean$^2$ corresponds to the conventional Gamma GLM, in which larger expected responses tend to exhibit proportionally larger variability. In contrast, the variance structure var *∝ * mean represents positive continuous outcomes whose absolute variability increases with the mean while relative variability decreases, as may occur in healthcare cost data [31]. One possible explanation is that relative variability decreases among higher-cost patients because patients with substantial healthcare needs tend to consume a more consistent level of services, such as ongoing medication use and routine monitoring.

We also vary the joint specification of exposures and adjustors. Each simulated dataset includes an intercept, a uniformly distributed adjustor *Z*, and a categorical exposure *X*. To capture realistic dependence, we use copula-based methods to introduce controlled correlation *ρ * between *X* and *Z* (see Section S.4 in the Supplemental Materials for details). This setup allows us to investigate how different dependence patterns between the exposure and the adjustor affect estimation and inference. Across all cases, we use a consistent simulation framework by specifying target type I error rate *α = 0.05* and power *q = 0.8*. Given *α *, *q* and degrees of freedom *p*, we compute the true non-centrality parameter *Δ * by solving equation (11). The link function and linear predictor are:

16 $$\begin{aligned} g(\mu ) = g(\mathbb {E}[Y \mid Z, D_1, D_2]) = \eta = \lambda _1 + \lambda _2 Z + \beta _1 D_{1} + \beta _2 D_{2}, \end{aligned}$$

where $X \in \{0,1,2\}$ is represented by two dummy variables $D_{1}$ and $D_{2}$. Let $\boldsymbol{\lambda } = (\lambda _1, \lambda _2)'$ and $\boldsymbol{\beta } = (\beta _1, \beta _2)'$. Consider the null hypothesis $H_0 : \boldsymbol{\beta }^* = \boldsymbol{0}$. Hence, the degrees of freedom *p = 2*, and the true non-centrality parameter needed to achieve 80% power is $\Delta \approx 9.63$.

### Wald test results

Given the joint distribution of *Z* and $(D_1,D_2)$ and the coefficients in (16), we compute the true asymptotic effect $f^{2}$ and the approximations $f^{2}_{\phi }$ and $f^{2}_R$. Since these do not admit closed-form expressions, we approximate them via Monte Carlo, by sampling *Z* and $(D_1,D_2)$ into a large data set and computing the associated *w* and $\eta _{x|z}$. The required sample sizes are then $n = \lceil \Delta / f^{2} \rceil$, $n_{\phi } = \lceil \Delta / f^{2}_{\phi } \rceil$, and $n_R = \lceil \Delta / f^{2}_R \rceil$, where $\lceil \cdot \rceil$ denotes the ceiling function. Let $N \in \{n, n_{\phi }, n_R\}$ be the chosen sample size. For each replicate, we generate an i.i.d. sample $\{(Y_i, Z_{0i}, D_{1i},D_{2i})\}_{i=1}^N$, where $Z_{0i}$ and $(D_1,D_2)$ follow Section S.4 in the Supplemental Materials, and outcome $Y_i$ is then generated based on the specified case. In the following, we set $\boldsymbol{Z} = (1, Z)$, and $\boldsymbol{X}=(D_{1}, D_{2})$ for each observation. The Wald statistic $\widehat{W}$ is computed, and $H_0$ is rejected if $\widehat{W} > F^{-1}_{\chi ^2_p(0)}(1 - \alpha )$. Repeating this procedure 10,000 times yields the empirical type I error rate under the null or the empirical power under the alternative, calculated as the rejection proportion.

#### Count outcomes

We investigate three cases of outcome distributions when the outcome is a counted quantity. **First**, $Y_i \sim \text {Poisson}(\mu _i)$, labeled “var = mean", which corresponds to a correctly specified GLM. The remaining two cases depart from the Poisson mean–variance relationship and are analyzed under a QL framework. **Second**, $Y_i \sim (1 - B_i) \text {Poi}(\mu _i) + B_i\text {Poi}(L_i)$, labeled “var *∝ * mean", where $B_i \sim \text {Bernoulli}(0.5)$ and $L_i \sim \text {Poi}(\mu _i)$ are mutually independent for each *i*. The outcome $Y_i$ is generated conditionally on $(B_i, L_i)$. **Third**, labeled “var *∝ * mean$^2$", outcomes follow a modified NB distribution, $Y_i \sim \text {NB}(\mu _i,\nu _i)$ with conditional mean and variance are $\mathbb {E}[Y_i \mid Z_i, D_{1i}, D_{2i}] = \mu _i$ and $\text {Var}(Y_i \mid Z_i, D_{1i}, D_{2i}) = \mu _i + \nu _i \mu _i^2$. We choose $\nu _i$ such that $\text {Var}(Y_i \mid Z_i, D_{1i}, D_{2i}) = \sigma ^2 \mu _i^2$, placing this setting within the class of models with a constant coefficient of variation (see Chapter 8 of McCullagh and Nelder [23]), where the variance is proportional to the square of the mean. Throughout the case of count outcomes, we fix $\sigma ^2 = 2$. This specification yields a non-standard NB distribution for which the maximum likelihood estimator (MLE) is difficult to obtain in closed form. Instead, the estimation of $(\boldsymbol{\lambda }, \boldsymbol{\beta })$ and the dispersion parameter $\sigma ^2$ follows the procedure in Section S.6 in the Supplemental Materials.

##### Log Link

**Fig. 1:**
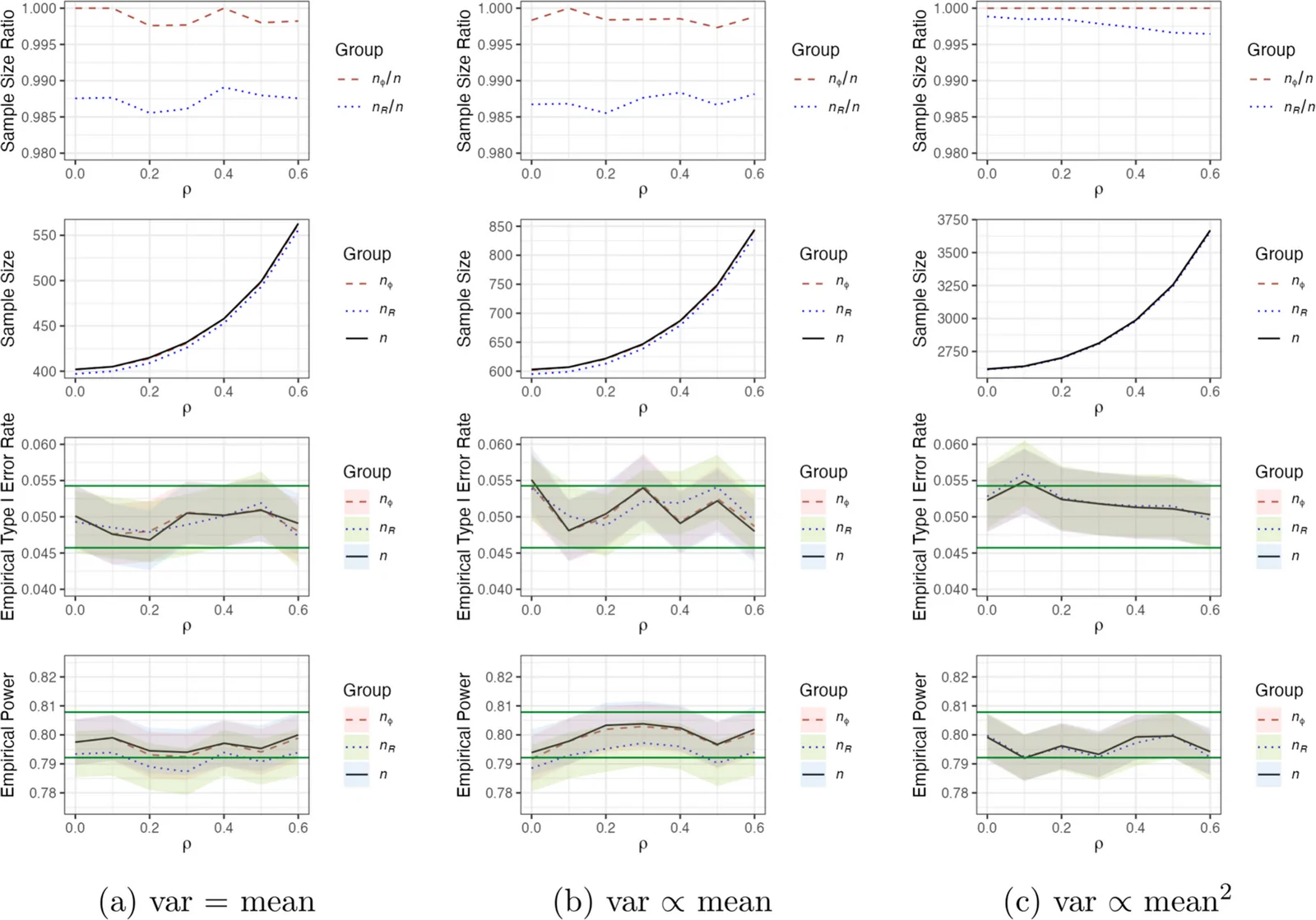
Empirical type I error rates and power of the Wald test based on simulated count data for three cases of outcome variables: **a** var = mean, **b** var *∝* mean, **c** var *∝* mean^2^. Results are shown for sample sizes *n*, $n_{\phi }$, and $n_{R}$, using a log link

With targeted type I error rate *α = 0.05* and power *q = 0.8*, we set the coefficients $\lambda _1 = 1, \lambda _2 = 0.15, \beta _1 = 0.1$, and $\beta _2 = 0.25$ in this simulation study. We use the log link function, so that $\frac{d\mu }{ d \eta } = \mu = \exp (\eta )$. The coefficients are chosen to achieve a target sample size of *n = 400* approximately under *ρ = 0* (see Section S.5 in the Supplemental Materials for details). Figure 1 presents the sample sizes *n*, $n_{\phi }$, and $n_{R}$ for three cases of outcome variable under this specification across varying values of the correlation parameter *ρ *, along with the corresponding empirical type I error rates and power, obtained from simulations. As * ρ * increases, both $\text {Var}(\eta _{x|z})$ and the pseudo-partial R-squared $R^{2}_{x|z}$ decline, because the unique contribution of $\boldsymbol{X}$ to the outcome * Y *, beyond that of $\boldsymbol{Z}$, diminishes as the dependence between the exposure $\boldsymbol{X}$ and the adjustor $\boldsymbol{Z}$ strengthens. Consequently, larger sample sizes are needed to maintain nominal power, a pattern consistent across all variance structures. Nevertheless, the impact of * ρ * on the ratios $n_{\phi }/n$ and $n_R/n_{s}$ is relatively minor, indicating that both 2SLiP and P2R2 remain robust for relative sample size estimation under varying dependence between exposures ($\boldsymbol{X}$) and adjustors ($\boldsymbol{Z}$).

Across all three cases, the 2SLiP-based sample size $n_{\phi }$ closely matches the true * n *. Notably, in the third case, where $\text {Var}(Y \mid \boldsymbol{Z}, \boldsymbol{X}) = \sigma ^2 \mu ^2$, the resulting weight * w * in (2) is constant, yielding $n_{\phi } = n$. This underscores the theoretical validity of 2SLiP in settings where the weight is constant. In contrast, the P2R2 approximation $R^{2}_{x|z}$ tends to overestimate the effect size $f^2$, and hence to underestimate the required sample size in the first two cases—“var = mean” and “var * ∝ * mean”—resulting in smaller values of $n_R$. Consequently, the achieved empirical power is lower than the nominal target, highlighting a potential limitation of P2R2 in such settings where the link function is far from linear or the variance structure is not conducive to the P2R2 approximation.

##### Identity Link

**Fig. 2:**
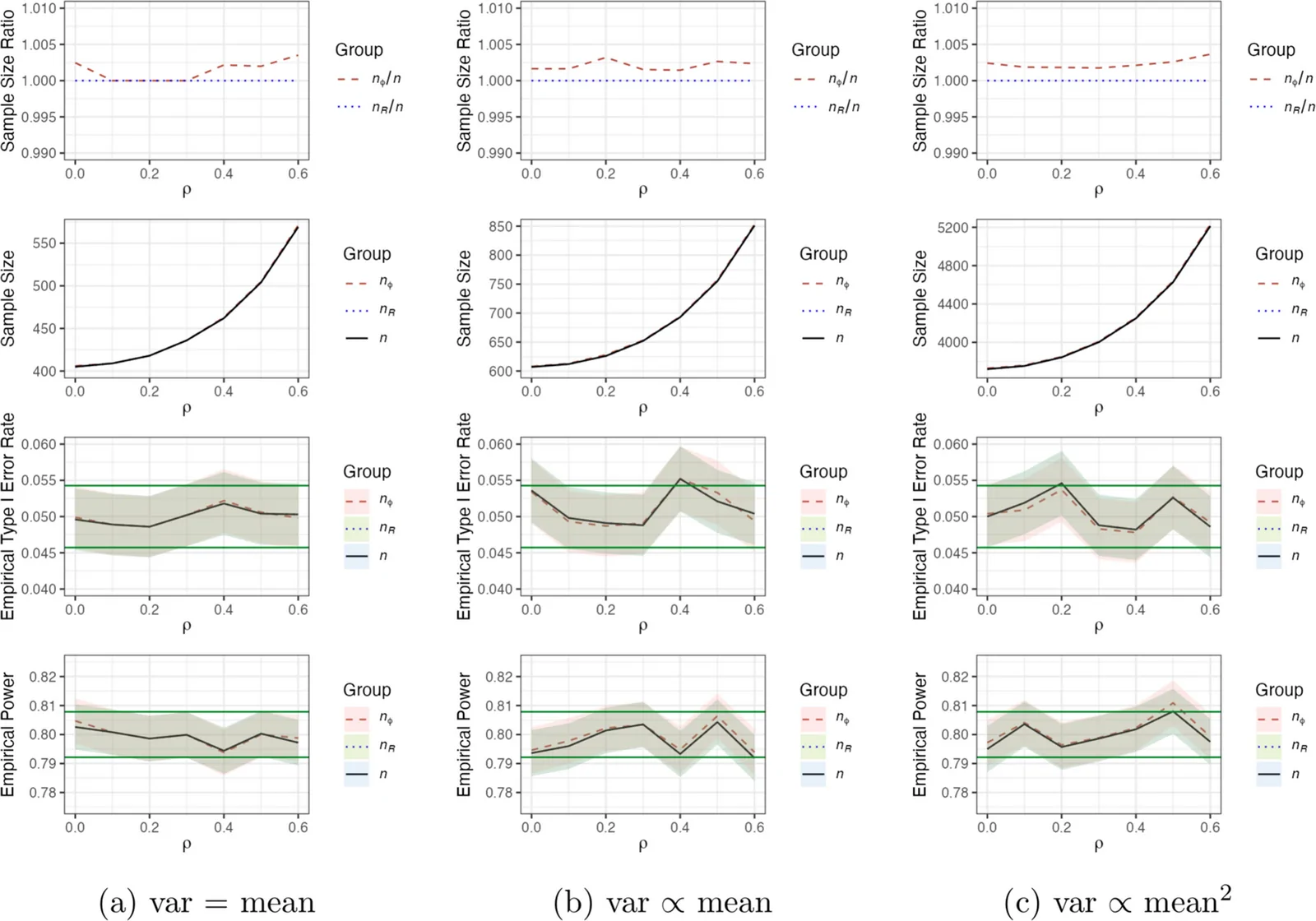
Empirical type I error rates and power of the Wald test based on simulated count data for three cases of outcome variables: **a** var = mean, **b** var *∝* mean, **c** var *∝ * mean^2^. Results are shown for sample sizes *n*, $n_{\phi }$, and $n_{R}$, using a log link

For the identity link, we set the coefficients $\lambda _1 = 4, \lambda _2 = 0.4, \beta _1 = 0.4$, and $\beta _2 = 0.81$, giving $\frac{d\mu }{ d \eta } = 1$. Figure 2 presents the sample sizes *n*, $n_{\phi }$, and $n_{R}$ for three cases of outcome variables across values of *ρ *, along with the empirical type I error rates and power.

Across all three cases and values of * ρ *, the 2SLiP-based sample size $n_{\phi }$ remains close to the true * n *, demonstrating robustness. Moreover, the P2R2-based sample size $n_{R}$ equals to * n * exactly, consistent with the definition of $f^{2}_{R}$ and its approximation condition in (13), which requires the link function * g(· ) * to be approximately linear. Under the identity link, $\mu = \eta$ and $\mu _z = \eta _z$, so *w = 1/v*, leading to $f^{2}_R = \mathbb {E}[(\mu - \mu _z)^2 / v] = \mathbb {E}[w \eta _{x|z}^2] = f^2$.

#### Positive continuous outcomes

We investigate two cases of outcome distributions when the outcome is a positive continuous quantity. **First**, labeled “var *∝ * mean", $Y_i \sim \text {Gamma}(\mu _i/\sigma ^2, \sigma ^2)$, where the second parameter $\sigma ^2$ is the scale. This specification yields mean $\mathbb {E}[Y_i \mid \boldsymbol{Z}_i, \boldsymbol{X}_i] = \mu _i$ and variance $\text {Var}(Y_i \mid \boldsymbol{Z}_i, \boldsymbol{X}_i) = \sigma ^2 \mu _i$. Estimation of $(\boldsymbol{\lambda }, \boldsymbol{\beta })$ and $\sigma ^2$ follows Section S.6 in the Supplemental Materials. **Second**, labeled “var *∝ * mean$^2$", assumes $Y_i \sim \text {Gamma}(1/\sigma ^2, \mu _i\sigma ^2)$, where the second parameter $\mu _i\sigma ^2$ is the scale. This specification yields mean $\mathbb {E}[Y_i \mid \boldsymbol{Z}_i, \boldsymbol{X}_i] = \mu _i$ and variance $\text {Var}(Y_i \mid \boldsymbol{Z}_i, \boldsymbol{X}_i) = \sigma ^2 \mu _i^2$, which is a standard GLM.

**Fig. 3:**
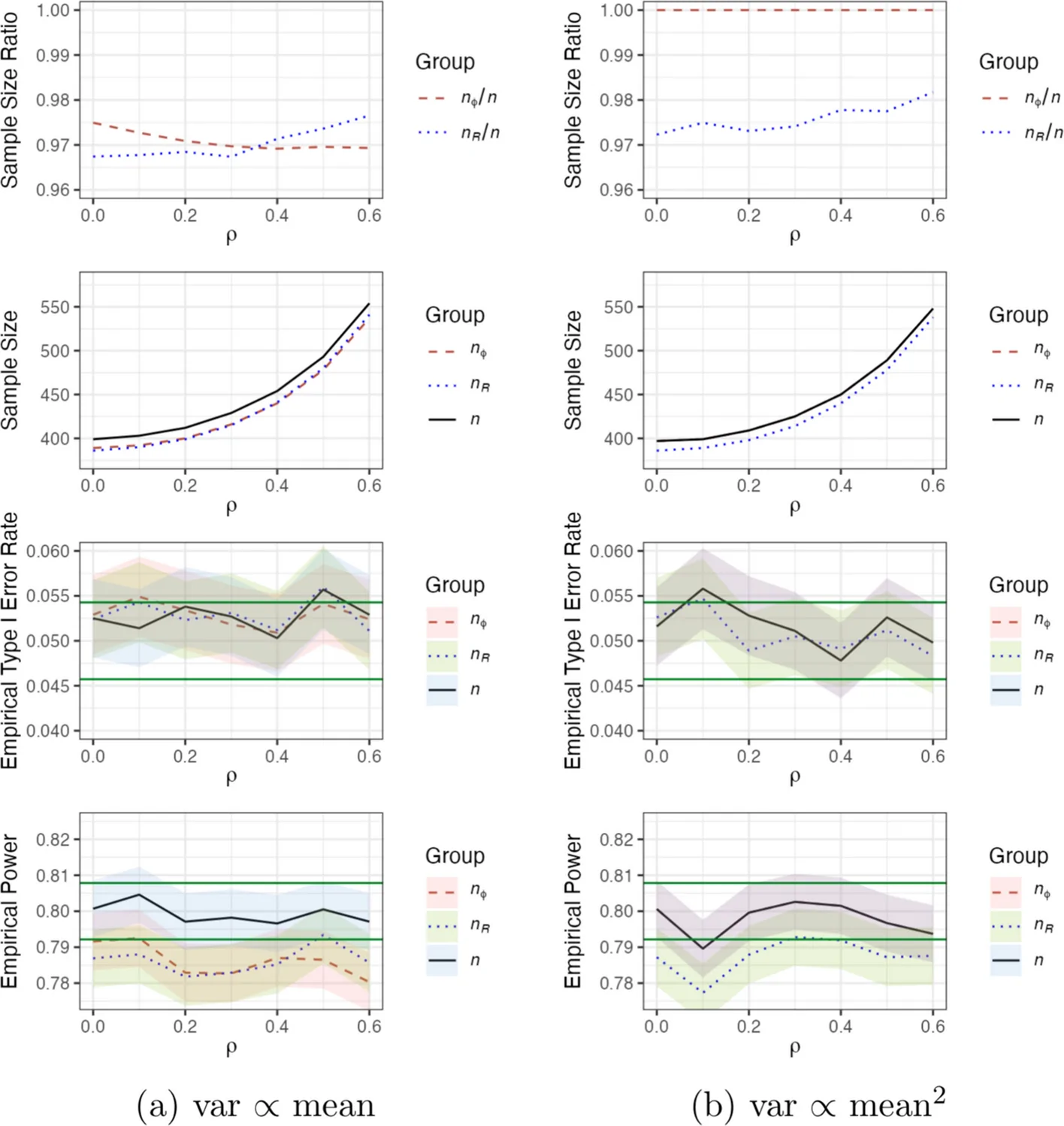
Empirical type I error rates and power of the Wald test based on simulated positive continuous data for two cases of outcome variables: **a** var *∝* mean, **b** var *∝* mean^2^. Results are shown for sample sizes *n*, $n_{\phi }$, and $n_{R}$, using a log link

With targeted type I error rate *α = 0.05* and power *q = 0.8*, we set the coefficients $\lambda _1 = 1, \lambda _2 = 0.15, \beta _1 = 0.1$, and $\beta _2 = 0.15$ in this simulation study. The dispersion parameter is set to $\sigma ^2 = 0.5$ for the first case and $\sigma ^2 = 0.16$ for the second, both under a log link. Figure 3 presents the sample sizes *n*, $n_{\phi }$, and $n_{R}$ across values of *ρ *, together with the corresponding empirical type I error rates and power.

As seen in the foregoing study, increasing *ρ * reduces the unique contribution of $\boldsymbol{X}$ beyond $\boldsymbol{Z}$, thereby increasing the required sample size. In the “var *∝ * mean” case (Fig. 3a), both $n_{\phi }$ and $n_{R}$ slightly underestimate *n*, resulting in modest under-powering. By contrast, in the “var *∝ * mean$^2$" case (Fig. 3b), only $n_{R}$ slightly underestimates *n*, while $n_{\phi }$ matches *n* exactly. This exact equality arises for the same reason as in the third case of the log-link count outcome: when $\text {Var}(Y \mid \boldsymbol{Z}, \boldsymbol{X}) = \sigma ^2 \mu ^2$, the resulting weight * w * is constant.

Across all link–variance combinations, the ratio of approximate to the true sample size deviated from unity by no more than 3%, with the largest under-estimation $(n_R/n_{s} \approx 0.97)$ occurring for P2R2 in the “var *∝ * mean, log-link” positive continuous case. Overall, these results collectively support the conclusion that 2SLiP and P2R2 offer consistently reliable performance for positive continuous outcomes. Regardless of the underlying variance structure, both measures provide valuable tools for PSS in GLMs and QL models.

We also conduct a parallel set of simulations in which $\beta _2$ is varied while the exposure–adjustor correlation is fixed at a moderate level. These simulations provide a direct sensitivity check of how changes in effect magnitude translate into effect size and required sample size. The outcome distributions, data-generating mechanisms, estimation procedures, and Wald-test implementation are identical to those described above, and the results reinforce the robustness patterns observed in the main simulation grid (see Supplementary Section S.7).

### Score test results

Following the setting of the simulation and in Wald test results section, we target target type I error rate *α = 0.05* and power *q = 0.8*. Given *α *, *q*, and degrees of freedom *p*, the true non-centrality parameter *Δ * is obtained by solving equation (11). We then link *Δ * to the approximate score-test effect $f_s^2$ to compute the required sample size. For a given data-generating mechanism (for example, as described at the beginning of Simulations section), $f_s^2$ is evaluated by Monte Carlo: draw a large i.i.d. sample of $(Y,Z,D_1,D_2)$ under known $(\boldsymbol{\lambda }, \boldsymbol{\beta })$, obtain $\widehat{\boldsymbol{\lambda }}^{(0)}$ and $\widehat{w}^{(0)}$, and compute the sample analogue of $f_s^2$ from (15). The required sample size is then $n_s = \lceil \Delta / f_s^2\rceil$, where $\lceil \cdot \rceil$ denotes the ceiling function. For comparison, we also report $n_{\phi } = \lceil \Delta / f^{2}_{\phi } \rceil$ and $n_R = \lceil \Delta / f^{2}_{R} \rceil$. Throughout this section we use $\lambda _1 = 1, \lambda _2 = 0.15, \beta _1 = 0.1$, and $\beta _2 = 0.21$ and the log link.

Having set up and computed the required values for each case, let $N \in \{n_s, n_{\phi }, n_R\}$ be the chosen sample size. For each replicate, we generate an i.i.d. sample $\{(Y_i, Z_{i}, D_{1i}, D_{2i})\}_{i=1}^N$ where $Z_{i}$ and $(D_{1i}, D_{2i})$ follow Section S.4 in the Supplemental Materials, and the count outcome $Y_i$ is drawn from the distributions in Count outcomes section, labled “var = mean", “var *∝ * mean", and “var *∝ * mean$^2$". For each replicate and sample size, we compute the score statistic $\widehat{S}$ If $\widehat{S} > F^{-1}_{\chi ^2_p(0)}(1 - \alpha )$, we reject $H_0$; otherwise, we do not. Repeating this procedure 10,000 times yields the empirical type I error rate under the null or the empirical power under the alternative, calculated as the proportion of simulations rejecting $H_0$.

**Fig. 4:**
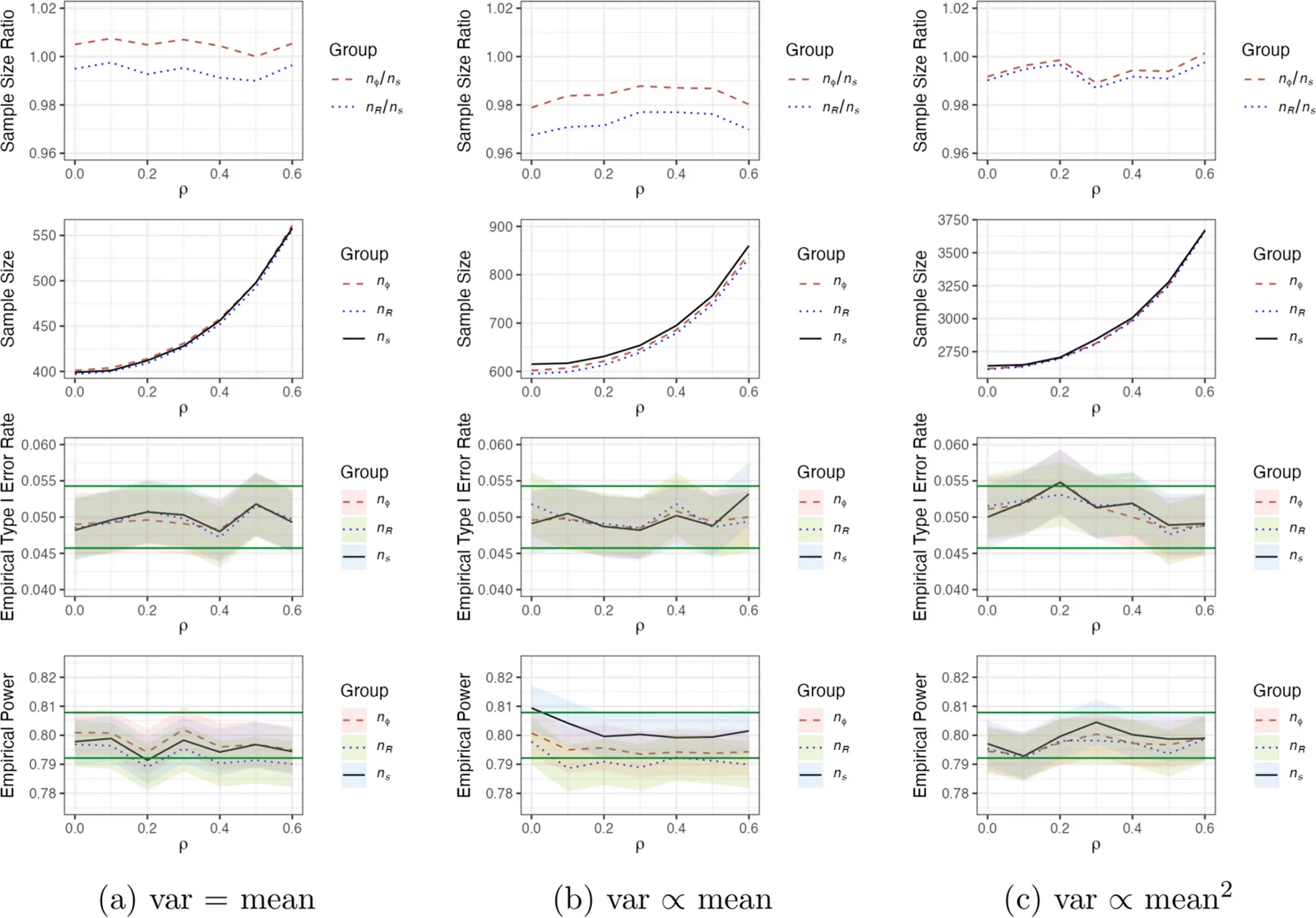
Empirical type I error rates and power of the score test based on simulated count data for three cases of outcome variables: **a** var = mean, **b** var *∝* mean, **c** var *∝* mean^2^. Results are shown for sample sizes *n*, $n_{\phi }$, and $n_{R}$, using a log link

Figure 4 presents the sample sizes $n_s$, $n_{\phi }$, and $n_{R}$ for three outcome cases across values of the correlation parameter *ρ *, along with the empirical type I error rates and power. Across all cases, the realized type I error remained within the target 0.05±0.004 envelope, confirming that the score statistic is well calibrated. The sample size $n_s$ (from the approximated score-test effect $f_s^2$) served as the benchmark. Both $n_{\phi }$ and $n_{R}$ matched $n_s$ closely for “var *=* mean” and “var *∝ * mean$^2$”, with at most 1% deviation. For “var *∝ * mean”, the largest underestimation was 3% ($n_{\phi }/n_s = 0.98$, $n_{R}/n_s = 0.97$). Increasing *ρ * (stronger exposure–adjustor association) inflated all three required sample sizes, but did not magnify the approximation error: the ratios $n_{\phi }/n_s$ and $n_{R}/n_s$ remained flat across the correlation grid. These findings mirror those obtained for the Wald test in Wald test results section, underscoring that 2SLiP and P2R2 transfer seamlessly from Wald to score tests.

## Case study

We illustrate the two approximated effect size measures, and accompanying PSS calculations, using survey data collected by Cahill et al. [24] from frontline health care workers during the COVID-19 pandemic. We first analyze the data and then consider a case where these data are viewed as arising from a pilot study and show how they can be used for PSS for a future study. The dataset includes responses from participants across two health systems in central Texas and features the 23-item Burnout Assessment Tool (BAT-23) as a measure of burnout risk. Along with burnout outcomes, participants reported their perceptions of personal protective equipment (PPE) adequacy and provided demographic and occupational information, offering a comprehensive basis for evaluating workplace factors and mental health. In this section, we first estimated the regression coefficients, then computed the two approximated effect size measures (2SLiP and P2R2), and finally examined how variation in the hypothetical regression coefficients for the exposure influences both the effect size and the sample size requirements for future studies planned for similar designs and instruments, to be analyzed within the QL framework.

Within this case study, we applied the QL framework to model the association between perceived PPE adequacy and burnout scores. The QL approach requires only the specification of the linear predictor, link function, and variance function, making it particularly appropriate for survey-based mental health data where distributional assumptions are uncertain. In our analysis, BAT-23 scores were treated as a positive continuous outcome *Y* and modeled with a log link $g(\cdot ) = \log (\cdot )$ under a variance structure proportional to the mean. As model comparison is beyond the scope of this manuscript, we adopted this model framework (log link and “var *∝ * mean") only because it does not guarantee exact effect size computation under either 2SLiP or P2R2, thereby allowing us to simultaneously illustrate their robustness. Perceived PPE adequacy was entered as the primary exposure $\boldsymbol{X}$, with demographic and occupational covariates (age, gender, education, and role) included as adjustors $\boldsymbol{Z}$ in the model. The exposure and adjustors were all categorical variables and were therefore represented using dummy variables in the regression. Specifically, perceived PPE adequacy was categorized as *Very Confident*, *Somewhat Confident*, *Uncertain*, *Somewhat Doubtful*, and *Very Doubtful*. *Very Confident* was treated as the reference category, and four dummy variables were created accordingly, resulting in four degrees of freedom for this exposure.

In this model specification, the log of the conditional mean of the outcome is given by $\log (\mathbb {E}[Y_i \mid \boldsymbol{Z}_i, \boldsymbol{X}_i]) = \log (\mu _i) = \boldsymbol{\lambda }'\boldsymbol{Z}_i + \boldsymbol{\beta }'\boldsymbol{X}_i$, with variance specified as $\text {Var}(Y_i \mid \boldsymbol{Z}_i, \boldsymbol{X}_i) = \sigma ^2 \mu _i$. Using the estimation procedure described in Section S.6 in the Supplemental Materials, we obtained the estimated regression coefficients for the exposure

$$\begin{aligned} \begin{array}{ccccccc} \widehat{\boldsymbol{\beta }} = & ( & 0.064, & 0.100, & 0.185, & 0.142 & )', \\ & & {Somewhat} & Uncertain & Somewhat & Very\\ & & Confident & & Doubtful & Doubtful & \\ \end{array} \end{aligned}$$

along with estimates for the adjustors, $\widehat{\boldsymbol{\lambda }}$, and the dispersion parameter, $\widehat{\sigma ^2} = 0.217$. The elements of $\widehat{\boldsymbol{\beta }}$ represent the log-mean differences in burnout scores relative to the *Very Confident* group. Overall, the estimated coefficients $\widehat{\boldsymbol{\beta }}$ indicate that lower perceived PPE adequacy is associated with higher expected burnout scores, even after adjusting for demographic and occupational covariates.

Taking $\widehat{\boldsymbol{\lambda }}$ and $\widehat{\boldsymbol{\beta }}$ as the true values of $\boldsymbol{\lambda }$ and $\boldsymbol{\beta }$, and the empirical distribution from the data as the true distribution, we could compute $\phi _{x|z} = 0.096$, $R^{2}_{x|z} = 0.020$, the true effect size $f^2 = 0.022$ from (8). On the original burnout scale, $\exp (\phi _{x|z}) = 1.100$ represents the expected relative change of the burnout score when perceived PPE adequacy shifts from relatively “high” confidence (e.g., *Very Confident*) to relatively “low” confidence (e.g., *Very Doubtful*), averaged over how workers are actually distributed across the adequacy categories and adjusting for other covariates. $R^{2}_{x|z}$ quantifies how much better the model predicts burnout when perceived PPE adequacy is included, beyond what’s already explained by demographic and occupational covariates. Here, $R^{2}_{x|z} = 0.020$ implies that perceived PPE adequacy explains 2.0% of the variation in burnout scores that is not already accounted for by demographics and occupational role. Finally, $\phi _{x|z} = 0.096$ and $R^{2}_{x|z} = 0.020$ correspond to two approximated effect sizes $f^2_\phi = 0.020$ and $f^2_R = 0.021$, both of which slightly underestimate the true effect size $f^2$.

**Fig. 5:**
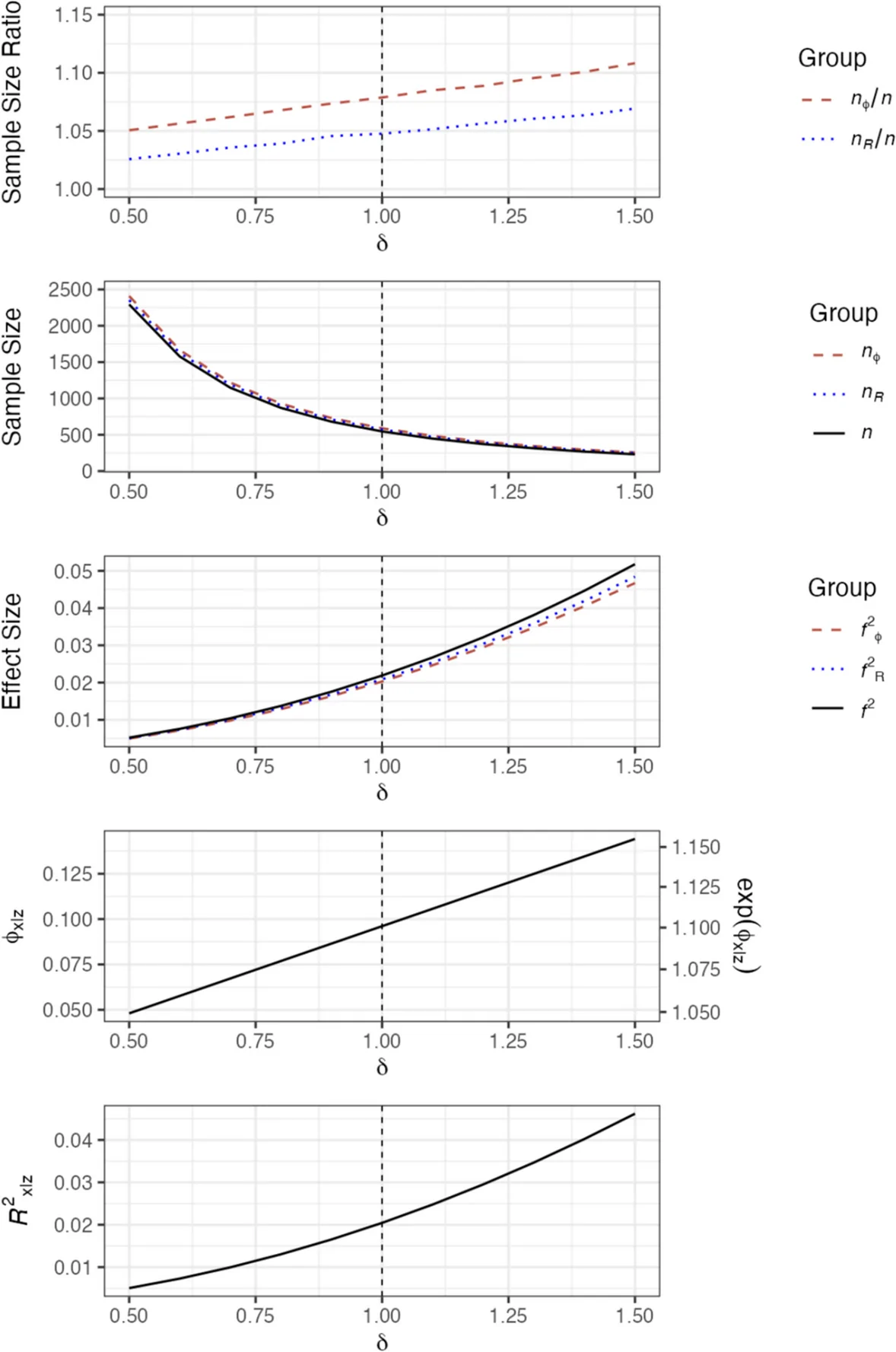
Performance of effect size measures and corresponding sample sizes across scaled exposure effects

Building on this, and to show how these base values might be used as reference pilot values in study planning, we fixed $\widehat{\boldsymbol{\lambda }}$ as the true value of $\boldsymbol{\lambda }$ and set $\delta \cdot \widehat{\boldsymbol{\beta }}$ as the true value of $\boldsymbol{\beta }$, varying *δ * from 0.5 to 1.5 to evaluate the performance of $\phi _{x|z}$ and $R^{2}_{x|z}$. With type I error rate *α = 0.05* and degrees of freedom *p = 4*, the true non-centrality parameter required to achieve 80% power is $\Delta \approx 11.94$. For each value of *δ *, we computed the true effect size $f^2$, the approximations $\phi _{x|z}$ and $R^{2}_{x|z}$ (with corresponding $f^2_\phi$ and $f^2_R$), and the approximate sample sizes required to achieve 80% power. These sample sizes were calculated as $n = \lceil \Delta / f^{2} \rceil$, $n_{\phi } = \lceil \Delta / f^{2}_{\phi } \rceil$, and $n_R = \lceil \Delta / f^{2}_R \rceil$. The results are presented in Fig. 5. The top panel of Fig. 5 shows the ratios $n_\phi /n$ and $n_R/n$, comparing sample sizes based on the approximations to those from the true effect size. Both ratios remain close to one, with deviations typically within 2–10% and rarely exceeding 10%. For example, when *δ = 1*, the required sample sizes are on the order of 500–600, with $n_\phi /n = 1.079$ and $n_R/n = 1.048$, showing that both approximations remain very close to the true requirement; in this case, the approximations slightly over-estimate the needed sample size, yielding conservative study designs. Importantly, the largest deviations occur when the true required sample size is relatively small (around 200), where even moderate differences in absolute numbers translate into larger percentage discrepancies.

Overall, the case study demonstrates that both $\phi _{x|z}$ and $R^{2}_{x|z}$ yield effect size approximations that closely track the true $f^2$, providing robust and practically reliable guidance for sample size determination under the quasi-likelihood framework.

## Discussion

Using extensive simulations that span canonical and non–canonical GLMs and QL models, we found that the two effect–size measures, 2SLiP and P2R2, retain their accuracy for the Wald test, and, under more limited conditions, for the score test as well. The 2SLiP approximation is essentially exact whenever the weight is constant (e.g.,“var *∝ * mean$^{2}$"), while P2R2 becomes exact when the link function is linear (e.g., identity link). Even in deliberately unfavourable configurations, in our simulation investigations, both measures missed the target sample size by no more than 3%, and the empirical power achieved by both measures deviated from the target power by no more than 2%.

Traditional power procedures for GLMs either require full specification of the joint $(Y,\boldsymbol{X},\boldsymbol{Z})$ distribution [7, 9, 12] or rely on design-matrix enumerations that become unwieldy with many exposures [10]. By contrast, 2SLiP and P2R2 need only first- and second-moment information, inherit the intuitive interpretation of effect in the linear predictor and variance explained from classical regression, and apply unchanged to quasi-likelihood models with dispersion. These advantages make the new measures that we propose especially attractive when the data-generating mechanism is incompletely specified or the design involves many exposures.

We note that soliciting 2SLiP and P2R2 inputs from investigators can still pose a challenge. Investigators must form expectations about the partial contribution of the exposure $\boldsymbol{X}$ to the outcome, conditional on $\boldsymbol{Z}$. Additionally, both 2SLiP and P2R2 are influenced by the correlation between $\boldsymbol{X}$ and $\boldsymbol{Z}$ and by the variance structure of *Y*, further complicating direct elicitation. However, this difficulty is no greater than that of eliciting $R^2$ or Cohen’s *d* in linear regression settings, which similarly require investigators to anticipate variance explained by exposures that may not yet be fully characterized. To facilitate adoption, we are developing a user-friendly implementation of these methods as a web-based R Shiny application (https://glmpower.shinyapps.io/shinyapp/). A follow-up manuscript describing the software resource will also include discussions and examples related to the solicitation of 2SLiP and P2R2.

Our investigation is limited to balanced designs with categorical exposures of up to three levels. Extending the framework to highly unbalanced cells, continuous-by-continuous interactions, and mixed-effects GLMs—where random effects introduce additional variance components—remains an important direction for future work. Addressing these settings will further broaden the practical scope of 2SLiP and P2R2 in planning modern, complex studies.

## Supplementary Information

Supplementary Material 1.

## Data Availability

This manuscript primarily relies on simulated data to illustrate and evaluate the proposed methods. The simulated datasets and the simulation algorithms will be made publicly available upon acceptance of the manuscript. The real-world case study data are not publicly available due to confidentiality and data-use restrictions; however, the case study is included for illustrative purposes only and does not affect the main methodological conclusions of the manuscript.
