# Supplementary material for: Robust power and sample size calculations in quasi-likelihood models: methods and practice
Source: BMC Med Res Methodol. 2026 Jul 21;26:221. doi: 10.1186/s12874-026-02940-y (PMC13670577; doi:10.1186/s12874-026-02940-y)
Supplement: Supplementary file 1 [file 12874_2026_2940_MOESM1_ESM.pdf]

Supplementary Materials for "Robust Power and Sample Size Calculations in Quasi-likelihood Models: Methods and Practice"

Shijie Yuan*, Amy Cochran†, and Paul Rathouz‡

May 28, 2026

*Department of Statistics and Data Sciences, The University of Texas at Austin, Austin, TX 78712, USA; Email: sj.yuan@austin.utexas.edu

†Department of Math, Department of Population Health Sciences, University of Wisconsin Madison, Madison, WI 53706, USA; Email: cochran4@wisc.edu

‡Department of Population Health, The University of Texas at Austin, Austin, TX 78712, USA; Corresponding Email: paul.rathouz@austin.utexas.edu

S.1 Asymptotic Justification of the Wald Statistic under Quasi-Likelihood

We begin by approximating the expected information matrix $\boldsymbol{i}_n$ with the marginal information matrix $\boldsymbol{\mathcal{I}}$. As $\widehat{\boldsymbol{\lambda}}$ and $\widehat{\boldsymbol{\beta}}$ are consistent estimators of $\boldsymbol{\lambda}^*$ and $\boldsymbol{\beta}^*$, and assuming that $(\boldsymbol{X}_i, \boldsymbol{Z}_i)$ are i.i.d., the Law of Large Number gives $\boldsymbol{i}_n\left(\widehat{\boldsymbol{\lambda}}, \widehat{\boldsymbol{\beta}}\right)/n \xrightarrow{p} \boldsymbol{\mathcal{I}}$, where the weight w in $\boldsymbol{\mathcal{I}}$ is evaluated at the true QL parameter values of $\boldsymbol{\lambda}^*$ and $\boldsymbol{\beta}^*$. Hence, by McCullagh (1983),

$$\sqrt{n}\left(\begin{bmatrix}\widehat{\boldsymbol{\lambda}}\\ \widehat{\boldsymbol{\beta}}\end{bmatrix} - \begin{bmatrix}\boldsymbol{\lambda}^*\\ \boldsymbol{\beta}^*\end{bmatrix}\right) \overset{A}{\sim} \mathcal{N}\left(\boldsymbol{0}, \boldsymbol{\mathcal{I}}^{-1}\right).$$

For $\widehat{\boldsymbol{\beta}}$, $\boldsymbol{i}_{n,\beta|\lambda}\left(\widehat{\boldsymbol{\lambda}}, \widehat{\boldsymbol{\beta}}\right)/n \xrightarrow{p} \boldsymbol{\mathcal{I}}_{\boldsymbol{\beta}|\boldsymbol{\lambda}}$ and $\sqrt{n}\left(\widehat{\boldsymbol{\beta}} - \boldsymbol{\beta}^*\right) \overset{A}{\sim} \mathcal{N}\left(\boldsymbol{0}, \boldsymbol{\mathcal{I}}_{\boldsymbol{\beta}|\boldsymbol{\lambda}}^{-1}\right)$, where $\boldsymbol{\mathcal{I}}_{\boldsymbol{\beta}|\boldsymbol{\lambda}}^{-1}$ is the lower right $p \times p$ block of $\boldsymbol{\mathcal{I}}^{-1}$ given by the Schur complement:

$$\boldsymbol{\mathcal{I}}_{\boldsymbol{\beta}|\boldsymbol{\lambda}} \equiv \mathbb{E}\left[w\boldsymbol{X}\boldsymbol{X}'\right] - \mathbb{E}\left[w\boldsymbol{X}\boldsymbol{Z}'\right]\mathbb{E}\left[w\boldsymbol{Z}\boldsymbol{Z}'\right]^{-1}\mathbb{E}\left[w\boldsymbol{Z}\boldsymbol{X}'\right].$$

For testing the null hypothesis $H_0 : \boldsymbol{\beta}^* = 0$, the Wald test statistic is given by

$$\widehat{W} = \widehat{\boldsymbol{\beta}}'\boldsymbol{i}_{n,\boldsymbol{\beta}|\boldsymbol{\lambda}}\left(\widehat{\boldsymbol{\lambda}}, \widehat{\boldsymbol{\beta}}\right)\widehat{\boldsymbol{\beta}},$$

which can be approximated as

$$\widehat{W} \approx n\widehat{\boldsymbol{\beta}}'\boldsymbol{\mathcal{I}}_{\boldsymbol{\beta}|\boldsymbol{\lambda}}\widehat{\boldsymbol{\beta}}.$$

Under the asymptotic normality of $\sqrt{n}(\widehat{\boldsymbol{\beta}} - \boldsymbol{\beta}^*) \xrightarrow{d} N(\boldsymbol{0}, \boldsymbol{\mathcal{I}}_{\boldsymbol{\beta}|\boldsymbol{\lambda}}^{-1})$, we write:

$$\widehat{\boldsymbol{\beta}} = \boldsymbol{\beta}^* + \frac{1}{\sqrt{n}}\boldsymbol{O}, \text{ where } \boldsymbol{O} \sim N(\boldsymbol{0}, \boldsymbol{\mathcal{I}}_{\boldsymbol{\beta}|\boldsymbol{\lambda}}^{-1}).$$

Substituting into $\widehat{W}$, we obtain:

$$\begin{aligned}\widehat{W} \approx & n\left(\boldsymbol{\beta}^* + \frac{1}{\sqrt{n}}\boldsymbol{O}\right)' \boldsymbol{\mathcal{I}}_{\boldsymbol{\beta}|\boldsymbol{\lambda}} \left(\boldsymbol{\beta}^* + \frac{1}{\sqrt{n}}\boldsymbol{O}\right) \\ = & n\left[\boldsymbol{\beta}^{*\prime}\boldsymbol{\mathcal{I}}_{\boldsymbol{\beta}|\boldsymbol{\lambda}}\boldsymbol{\beta}^* + \frac{2}{\sqrt{n}}\boldsymbol{\beta}^{*\prime}\boldsymbol{\mathcal{I}}_{\boldsymbol{\beta}|\boldsymbol{\lambda}}\boldsymbol{O} + \frac{1}{n}\boldsymbol{O}'\boldsymbol{\mathcal{I}}_{\boldsymbol{\beta}|\boldsymbol{\lambda}}\boldsymbol{O}\right] \\ = & n\boldsymbol{\beta}^{*\prime}\boldsymbol{\mathcal{I}}_{\boldsymbol{\beta}|\boldsymbol{\lambda}}\boldsymbol{\beta}^* + 2\sqrt{n}\boldsymbol{\beta}^{*\prime}\boldsymbol{\mathcal{I}}_{\boldsymbol{\beta}|\boldsymbol{\lambda}}\boldsymbol{O} + \boldsymbol{O}'\boldsymbol{\mathcal{I}}_{\boldsymbol{\beta}|\boldsymbol{\lambda}}\boldsymbol{O}.\end{aligned}$$

Take the expectation of $\widehat{W}$. The first term is deterministic; the second term has expectation zero since $\mathbb{E}[\boldsymbol{O}] = \mathbf{0}$; the third term evaluates to:

$$\mathbb{E}\left[\boldsymbol{O}'\boldsymbol{\mathcal{I}}_{\boldsymbol{\beta}|\boldsymbol{\lambda}}\boldsymbol{O}\right] = \operatorname{tr}\left(\boldsymbol{\mathcal{I}}_{\boldsymbol{\beta}|\boldsymbol{\lambda}}\mathbb{E}\left[\boldsymbol{O}\boldsymbol{O}'\right]\right) = \operatorname{tr}\left(\boldsymbol{\mathcal{I}}_{\boldsymbol{\beta}|\boldsymbol{\lambda}}\boldsymbol{\mathcal{I}}^{-1}_{\boldsymbol{\beta}|\boldsymbol{\lambda}}\right) = p.$$

Thus, the expectation of the Wald statistic is $\mathbb{E}\left[\widehat{W}\right] = n\boldsymbol{\beta}^{*\prime}\boldsymbol{\mathcal{I}}_{\boldsymbol{\beta}|\boldsymbol{\lambda}}\boldsymbol{\beta}^* + p$. This implies that the non-centrality parameter is $\Delta = n\boldsymbol{\beta}^{*\prime}\boldsymbol{\mathcal{I}}_{\boldsymbol{\beta}|\boldsymbol{\lambda}}\boldsymbol{\beta}^* = nf^2$, and the corresponding effect

$$f^2 = \boldsymbol{\beta}^{*\prime}\boldsymbol{\mathcal{I}}_{\boldsymbol{\beta}|\boldsymbol{\lambda}}\boldsymbol{\beta}^*.$$

S.2 Theoretical Justification of Effect Size–Based Power Approximations

The preceding derivation in Section S.1 establishes the asymptotic behavior of the Wald statistic from an information-based perspective; we next offer a complementary explanation by exploiting the representation of QLEs as solutions to iteratively weighted least squares. Specifically, The QLEs $\widehat{\boldsymbol{\lambda}}$ and $\widehat{\boldsymbol{\beta}}$ can be obtained from n independent observations of $(Y, \boldsymbol{X}, \boldsymbol{Z})$ by iteratively constructing a linearized working variable

$$g(Y) \approx Y_l \equiv \eta + \frac{d\eta}{d\mu}(Y - \mu),$$

and QLEs $(\widehat{\boldsymbol{\lambda}}, \widehat{\boldsymbol{\beta}})$ result from regressing Y_l onto $(\boldsymbol{X}, \boldsymbol{Z})$ with the weight w, updating until convergence. Equivalently, this can be written as

$$w^{1/2}Y_l = w^{1/2}\eta + \frac{(Y-\mu)}{\sqrt{v}} = \boldsymbol{\lambda}' w^{1/2}\boldsymbol{Z} + \boldsymbol{\beta}' w^{1/2}\boldsymbol{X} + \frac{(Y-\mu)}{\sqrt{\sigma^2 v}}. \tag{S.1}$$

To further disentangle the contributions of $\boldsymbol{X}$ and $\boldsymbol{Z}$, we regress $w^{1/2}\boldsymbol{X}$ on $w^{1/2}\boldsymbol{Z}$ to extract the residual component of $\boldsymbol{X}$ that is orthogonal to $\boldsymbol{Z}$ in the weighted space, leaving the residual $w^{1/2}(\boldsymbol{X} - \boldsymbol{A}\boldsymbol{Z})$, where $\boldsymbol{A}_{p\times r}$ denotes the population limit of the weighted least squares estimation of the regression coefficients from regressing $\boldsymbol{X}$ on $\boldsymbol{Z}$, defined as

$$\boldsymbol{A} = \mathbb{E}[w\boldsymbol{X}\boldsymbol{Z}']E[w\boldsymbol{Z}\boldsymbol{Z}']^{-1}. \tag{S.2}$$

Substituting this decomposition into (S.1) yields

$$w^{1/2}Y_l = w^{1/2}(\boldsymbol{\lambda}' + \boldsymbol{\beta}'\boldsymbol{A})\boldsymbol{Z} + w^{1/2}(\boldsymbol{\beta}'\boldsymbol{X} - \boldsymbol{\beta}'\boldsymbol{A}\boldsymbol{Z}) + \frac{(Y-\mu)}{\sqrt{\sigma^2 v}}. \tag{S.3}$$

As our focus is on interpretation rather than estimation, we specialize to the true QL parameter values $\boldsymbol{\lambda}^*$ and $\boldsymbol{\beta}^*$. In this case, the linear predictor admits the decomposition $\eta = \eta_z + \eta_{x|z}$, where

$$\eta_z = (\boldsymbol{\lambda}^{*\prime} + \boldsymbol{\beta}^{*\prime}\boldsymbol{A})\boldsymbol{Z} \tag{S.4}$$

captures the contribution of the adjustors $\boldsymbol{Z}$, and

$$\eta_{x|z} = \boldsymbol{\beta}^{*\prime}(\boldsymbol{X} - \boldsymbol{A}\boldsymbol{Z})$$

represents the unique contribution of the residualized $\boldsymbol{X}$, scaled by $w^{1/2}$. Consequently,

$$w^{1/2}Y_l = w^{1/2}\eta_z + w^{1/2}\eta_{x|z} + \frac{(Y-\mu)}{\sqrt{\sigma^2 v}}.$$

It follows that the true effect f^2 can be equivalently expressed as

$$f^2 = \boldsymbol{\beta}^{*\prime}\mathbb{E}[w(\boldsymbol{X} - \boldsymbol{A}\boldsymbol{Z})(\boldsymbol{X} - \boldsymbol{A}\boldsymbol{Z})']\boldsymbol{\beta}^* = \mathbb{E}\left[w\eta_{x|z}^2\right],$$

since

$$
\begin{aligned}
f^2 =& \boldsymbol{\beta}^{*\prime}\mathbb{E}[w(\boldsymbol{X}-\boldsymbol{A}\boldsymbol{Z})(\boldsymbol{X}-\boldsymbol{A}\boldsymbol{Z})']\boldsymbol{\beta}^* \\
=& \boldsymbol{\beta}^{*\prime}\mathbb{E}[w(\boldsymbol{X}\boldsymbol{X}'-\boldsymbol{X}\boldsymbol{Z}'\boldsymbol{A}'-\boldsymbol{A}\boldsymbol{Z}\boldsymbol{X}'+\boldsymbol{A}\boldsymbol{Z}\boldsymbol{Z}'\boldsymbol{A})]\boldsymbol{\beta}^* \\
=& \boldsymbol{\beta}^{*\prime}\left(\mathbb{E}[w\boldsymbol{X}\boldsymbol{X}']-\mathbb{E}[w\boldsymbol{X}\boldsymbol{Z}']\boldsymbol{A}'-\boldsymbol{A}\mathbb{E}[w\boldsymbol{Z}\boldsymbol{X}']+\boldsymbol{A}\mathbb{E}[w\boldsymbol{Z}\boldsymbol{Z}']\boldsymbol{A}'\right)\boldsymbol{\beta}^* \\
=& \boldsymbol{\beta}^{*\prime}\left(\mathbb{E}[w\boldsymbol{X}\boldsymbol{X}']-\mathbb{E}[w\boldsymbol{X}\boldsymbol{Z}']E[w\boldsymbol{Z}\boldsymbol{Z}']^{-1}\mathbb{E}[w\boldsymbol{Z}\boldsymbol{X}']\right)\boldsymbol{\beta}^* \\
=& \boldsymbol{\beta}^{*\prime}\boldsymbol{\mathcal{I}}_{\boldsymbol{\beta}|\boldsymbol{\lambda}}\boldsymbol{\beta}^*.
\end{aligned}
$$

The fourth equality holds because

$$
\mathbb{E}[w\boldsymbol{X}\boldsymbol{Z}']\boldsymbol{A}' = \boldsymbol{A}\mathbb{E}[w\boldsymbol{Z}\boldsymbol{X}'] = \boldsymbol{A}\mathbb{E}[w\boldsymbol{Z}\boldsymbol{Z}']\boldsymbol{A}' = \mathbb{E}[w\boldsymbol{X}\boldsymbol{Z}']E[w\boldsymbol{Z}\boldsymbol{Z}']^{-1}\mathbb{E}[w\boldsymbol{Z}\boldsymbol{X}'].
$$

Throughout, the expectation, variance, and covariance operators are taken over the joint distribution of $\boldsymbol{X}$ and $\boldsymbol{Z}$, with the weight w evaluated at the the true QL parameter values $\boldsymbol{\lambda}^*$ and $\boldsymbol{\beta}^*$.

Approximation of True Effect Expansion of $\mathbb{E}\left[w\eta_{x|z}^2\right]$ yields

$$
f^2 = \mathbb{E}[w]\mathbb{E}\left[\eta_{x|z}^2\right] + \text{cov}\left[w, \eta_{x|z}^2\right] = \mathbb{E}[w]\left(E[\eta_{x|z}]^2 + \text{Var}[\eta_{x|z}]\right) + \text{cov}\left[w, \eta_{x|z}^2\right].
$$

The first measure $\phi_{x|z}$ captures a 2SD change in the residualized linear predictor $\eta_{x|z}$. Under local conditions where $\mathbb{E}\left[\eta_{x|z}\right] \approx 0$, $\text{cov}\left[w, \eta_x^2\right] \approx 0$, and $\mathbb{E}[w] \approx w_{\mathbb{1}}$, the true effect f^2 can be approximated by $f_\phi^2 \equiv w_{\mathbb{1}}\phi_{x|z}^2/4 \approx f^2$, where $w_{\mathbb{1}}$ denotes the value of w when evaluated at the population mean response $\mu = \mathbb{E}[Y]$, marginally w.r.t $\boldsymbol{X}$ and $\boldsymbol{Z}$.

The connection between f^2 and $R_{x|z}^2$ becomes clear through a first-order approximation. For small differences between η and η_z,

$$
\mu - \mu_z \approx \frac{d\mu}{d\eta}(\eta - \eta_z),
$$

which implies $f_R^2 \equiv R^2_{x|z}/(1-R^2_{x|z}) \approx \mathbb{E}[w\eta^2_{x|z}] = f^2$. Thus $R^2_{x|z}$ connects directly to the non-centrality parameter in Wald tests, particularly when the link is nearly linear.

S.3 Equivalence of Reduced-Model Linear Predictors

The reduced-model linear predictor η_z is introduced from two perspectives: (i) as the solution to a weighted mean squared error (WMSE) minimization problem in (4) involving only the adjustors $\boldsymbol{Z}$, and (ii) as the component of the full linear predictor (S.4) obtained by partialling out $\boldsymbol{X}$ with respect to $\boldsymbol{Z}$ in the weighted space. Here we show that these two constructions are equivalent.

Recall that the reduced-model coefficients are defined as

$$\boldsymbol{\lambda}_0^* = \arg\min_{\boldsymbol{\lambda}} \mathbb{E}\left[w\{Y_l - \boldsymbol{\lambda}'\boldsymbol{Z}\}^2\right],$$

where the working variable Y_l and weight w are evaluated at the quasi-true parameter values $(\boldsymbol{\lambda}^*, \boldsymbol{\beta}^*)$ of the full model. The first-order condition for this minimization problem is

$$\mathbb{E}[w\boldsymbol{Z}\boldsymbol{Z}']\boldsymbol{\lambda} = \mathbb{E}[w\boldsymbol{Z}Y_l].$$

Under the working linearization at the true QL parameter values, the linearized outcome admits the decomposition

$$Y_l = (\boldsymbol{\lambda}^{*\prime} + \boldsymbol{\beta}^{*\prime}\boldsymbol{A})\boldsymbol{Z} + (\boldsymbol{\beta}^{*\prime}\boldsymbol{X} - \boldsymbol{\beta}^{*\prime}\boldsymbol{A}\boldsymbol{Z}) + \frac{d\eta}{d\mu}(Y-\mu).$$

To establish the equivalence, we substitute the above decomposition of Y_l into the first-order condition $\mathbb{E}[w\boldsymbol{Z}Y_l]$. By linearity of expectation, this yields three corresponding terms, which we examine in turn.

First term. Consider

$$\mathbb{E}\big[w\boldsymbol{Z}\big\{(\boldsymbol{\lambda}^{*\prime}+\boldsymbol{\beta}^{*\prime}\boldsymbol{A})\boldsymbol{Z}\big\}\big]=\mathbb{E}[w\boldsymbol{Z}\boldsymbol{Z}'](\boldsymbol{\lambda}^{*}+\boldsymbol{A}'\boldsymbol{\beta}^{*}).$$

Second term. Next, consider

$$\mathbb{E}\big[w\boldsymbol{Z}\big\{\boldsymbol{\beta}^{*\prime}\boldsymbol{X}-\boldsymbol{\beta}^{*\prime}\boldsymbol{A}\boldsymbol{Z}\big\}\big]=\mathbb{E}[w\boldsymbol{Z}\boldsymbol{X}']\boldsymbol{\beta}^{*}-\mathbb{E}[w\boldsymbol{Z}\boldsymbol{Z}']\boldsymbol{A}'\boldsymbol{\beta}^{*}.$$

By the definition of $\boldsymbol{A}$ in (S.2), it follows that

$$\mathbb{E}[w\boldsymbol{Z}\boldsymbol{X}']=\mathbb{E}[w\boldsymbol{Z}\boldsymbol{Z}']\boldsymbol{A}',$$

and hence this term is identically zero.

Third term. Finally, consider the contribution from the working error term,

$$\mathbb{E}\left[w\boldsymbol{Z}\frac{d\eta}{d\mu}(Y-\mu)\right].$$

Under the QL assumption $\mathbb{E}[Y \mid \boldsymbol{X},\boldsymbol{Z}]=\mu$, we have

$$\mathbb{E}\left[\frac{d\eta}{d\mu}(Y-\mu)\mid \boldsymbol{X},\boldsymbol{Z}\right]=0,$$

and therefore, by iterated expectation,

$$\mathbb{E}\left[w\boldsymbol{Z}\frac{d\eta}{d\mu}(Y-\mu)\right]=\boldsymbol{0}.$$

Combining the three components, we obtain

$$\mathbb{E}[w\boldsymbol{Z}Y_l]=\mathbb{E}[w\boldsymbol{Z}\boldsymbol{Z}'](\boldsymbol{\lambda}^{*}+\boldsymbol{A}'\boldsymbol{\beta}^{*}).$$

Substituting this expression into the first-order condition yields

$$\mathbb{E}[w\boldsymbol{Z}\boldsymbol{Z}']\boldsymbol{\lambda}=\mathbb{E}[w\boldsymbol{Z}\boldsymbol{Z}'](\boldsymbol{\lambda}^{*}+\boldsymbol{A}'\boldsymbol{\beta}^{*}),$$

and, assuming $\mathbb{E}[w\boldsymbol{Z}\boldsymbol{Z}']$ is nonsingular,

$$\boldsymbol{\lambda}_0^* = \boldsymbol{\lambda}^* + \boldsymbol{A}'\boldsymbol{\beta}^*.$$

Consequently, the reduced-model linear predictor satisfies

$$\eta_z = \boldsymbol{\lambda}_0^{*\prime}\boldsymbol{Z} = (\boldsymbol{\lambda}^{*\prime} + \boldsymbol{\beta}^{*\prime}\boldsymbol{A})\boldsymbol{Z},$$

which coincides with the representation obtained by partialling out $\boldsymbol{X}$ with respect to $\boldsymbol{Z}$ in the weighted space. This establishes the equivalence of the two constructions of η_z.

S.4 RHS Data Generation

To introduce a dependency between the continuous uniform variable Z and a categorical variable X with three levels $\{0, 1, 2\}$, we use a Gaussian copula. The steps for generating Z and X using a Gaussian copula are as follows:

1. Generate correlated normal variables (C_1, C_2) from the bivariate normal distribution with mean zero and covariance matrix Σ:

   $$(C_1, C_2) \sim N\left(\begin{bmatrix} 0 \\ 0 \end{bmatrix}, \Sigma\right),$$

   where

   $$\Sigma = \begin{bmatrix} 1 & \rho \\ \rho & 1 \end{bmatrix},$$

   and $\rho \in [-1, 1]$ is the correlation parameter between C_1 and C_2.

2. Transform C_1 and C_2 to two uniform variables via the cumulative distribution function (CDF) of the standard normal distribution $\Phi(\cdot)$:

$$U_1 = \Phi(C_1), \quad U_2 = \Phi(C_2).$$

3. Set $Z = U_1$.

4. Define the categorical variable X by the interval into which U_2 falls:

$$X = \begin{cases} 0 & \text{if} \quad U_2 \in [0, 1/3), \\ 1 & \text{if} \quad U_2 \in [1/3, 2/3), \\ 2 & \text{if} \quad U_2 \in [2/3, 1]. \end{cases}$$

S.5 Coefficient Search

Algorithm 1 Adjust β_2 to match target sample size $n^\star$

Require: Target non-centrality parameter Δ, target sample size $n^\star$, joint distribution of (Z, D_1, D_2), coefficients $\lambda_1, \lambda_2, \beta_1$, initial $\beta_2 \in (-\infty, \infty)$, tolerance ϵ, step size κ

Ensure: Estimated β_2 such that $n \approx n^\star$

while not converged **do**
    Compute effect size $f^2 = f^2(\lambda_1, \lambda_2, \beta_1, \beta_2)$ using Monte Carlo methods
    Compute $n = \frac{\Delta}{f^2}$
    if $|n - n^\star| < \epsilon$ **then**
        break
    else if $n > n^\star$ **then**
        $\beta_2 := \beta_2 - \kappa$
    else
        $\beta_2 := \beta_2 + \kappa$
    end if
end while
return β_2

S.6 Quasi-Likelihood Estimation Algorithm

For simplicity, we use $\boldsymbol{X}$ to represent all covariates $\boldsymbol{X}$ and $\boldsymbol{Z}$ in the main manuscript, and let $\boldsymbol{\beta}$ denote their corresponding coefficients in the estimation algorithm. The parameter σ^2 denotes the dispersion.

In quasi-likelihood theory (Mccullagh and Nelder, 1989), if conditional mean and variance of Y given $\boldsymbol{X}$ are $\mathbb{E}[Y \mid \boldsymbol{X}] = \mu$ and $\mathrm{Var}(Y \mid \boldsymbol{X}) = v$, then the quasi-likelihood function $Q(Y; \boldsymbol{\beta}, \sigma^2)$ satisfies

$$\frac{\partial Q(Y; \boldsymbol{\beta}, \sigma^2)}{\partial \mu} = \frac{Y - \mu}{v}$$

The quasi-score function is the derivative of the quasi-likelihood with respect to the parameter vector $\boldsymbol{\beta}$,

$$\boldsymbol{U}(\boldsymbol{\beta}, \sigma^2) = \frac{\partial Q(Y; \boldsymbol{\beta}, \sigma^2)}{\partial \boldsymbol{\beta}} = \frac{\partial Q(Y; \boldsymbol{\beta}, \sigma^2)}{\partial \mu} \frac{d\mu}{d\eta} \frac{\partial \eta}{\partial \boldsymbol{\beta}} = \frac{Y - \mu}{v} \frac{d\mu}{d\eta} \boldsymbol{X}$$

Given N observations $\{Y_i, \boldsymbol{X}_i\}$, we iteratively estimate the coefficients using weighted least squares to solve the quasi-score equation

$$\boldsymbol{U}_N(\boldsymbol{\beta}, \sigma^2) = \sum_{i=1}^{N} \frac{Y_i - \mu_i}{v_i} \left(\frac{d\mu}{d\eta} \bigg|_{\eta = \eta_i} \right) \boldsymbol{X}_i = 0. \tag{S.5}$$

During the iterative procedure, we update the dispersion parameter σ^2 using the Pearson estimate.

Algorithm 2 applies to the third case of count outcome in Section 3.1.1, which follows a modified negative binomial distribution. In this setting, the dispersion parameter σ^2 and the variance v_i are updated as follows:

$$\sigma^{2(t+1)} = \frac{1}{N - p} \sum_{i=1}^{N} \frac{(Y_i - \mu_i^{(t)})^2}{\left(\mu_i^{(t)}\right)^2} \quad \text{and} \quad v_i^{(t)} = \sigma^{2(t)} (\mu_i^{(t)})^2$$

This algorithm can be readily adapted to estimate the coefficients and dispersion parameter for the first case of positive continuous outcome in Section 3.1.2 by modifying the updates as follows:

$$\sigma^{2^{(t+1)}} = \frac{1}{N-p} \sum_{i=1}^{N} \frac{(Y_i - \mu_i^{(t)})^2}{\mu_i^{(t)}} \quad \text{and} \quad v_i^{(t)} = \sigma^{2^{(t)}} \mu_i^{(t)}$$

Algorithm 2 Iterative Quasi-Likelihood Estimation

Require: N observations $\{Y_i, \boldsymbol{X}_i\}$ (design matrix $\boldsymbol{X}_{(N)} = \begin{pmatrix} \boldsymbol{X}_1 & \dots & \boldsymbol{X}_N \end{pmatrix}'$), link function $g(\cdot)$, initial regression coefficients $\boldsymbol{\beta}^{(0)}$ and dispersion parameter $\sigma^{2(0)}$, tolerance ϵ

Ensure: Estimated $\boldsymbol{\beta}$ and σ^2 satisfying the quasi-score equation (S.5)

for iteration $t = 0, 1, 2, \dots$ **do**

  for each observation $i = 1, \dots, N$ **do**

    Compute linear predictor: $\eta_i^{(t)} = \boldsymbol{\beta}^{(t)\prime} \boldsymbol{X}_i$

    Compute mean: $\mu_i^{(t)} = g^{-1}(\eta_i^{(t)})$

    Compute variance: $v_i^{(t)} = \sigma^{2(t)} (\mu_i^{(t)})^2$

    Compute working variable:

$$z_i^{(t)} = \eta_i^{(t)} + (Y_i - \mu_i^{(t)}) \cdot \left(\frac{d\eta}{d\mu}\Big|_{\mu=\mu_i^{(t)}} \right)$$

    Compute weight:

$$w_i^{(t)} = \frac{1}{v_i^{(t)}} \left(\frac{d\mu}{d\eta}\Big|_{\eta=\eta_i^{(t)}} \right)^2$$

  end for

  Form weight matrix $\boldsymbol{W}^{(t)} = \mathrm{diag}(w_1^{(t)}, \dots, w_N^{(t)})$

  Form working response vector $\boldsymbol{z}^{(t)} = (z_1^{(t)}, \dots, z_N^{(t)})'$

  Update coefficients:

$$\boldsymbol{\beta}^{(t+1)} = \left(\boldsymbol{X}_{(N)}' \boldsymbol{W}^{(t)} \boldsymbol{X}_{(N)} \right)^{-1} \boldsymbol{X}_{(N)}' \boldsymbol{W}^{(t)} \boldsymbol{z}^{(t)}$$

  Update dispersion:

$$\sigma^{2(t+1)} = \frac{1}{N-p} \sum_{i=1}^{N} \frac{(Y_i - \mu_i^{(t)})^2}{\left(\mu_i^{(t)}\right)^2}$$

  if $\|\boldsymbol{\beta}^{(t+1)} - \boldsymbol{\beta}^{(t)}\|_\infty < \epsilon$ **and** $|\sigma^{2(t+1)} - \sigma^{2(t)}| < \epsilon$ **then**

    break

  end if

end for

S.7 Additional Simulation Results

To further examine the performance of 2SLiP and P2R2 on power and sample size estimation, we conducted an additional set of simulations, fixing the correlation parameter at $\rho = 0.3$ and varying the coefficient β_2 while holding other parameters constant. This setup isolates the impact of one exposure's effect size under a moderate degree of predictor–adjustor dependence. For each value of β_2, we computed the true non-centrality parameter f^2, its approximations f_ϕ^2 and f_R^2, and the corresponding required sample sizes n, n_ϕ, and n_R. The outcome distributions, data-generating mechanisms, estimation procedures, and Wald-test conduction mirror those described in Section 3.1.

S.7.1 Count Outcomes

S.7.1.1 Log Link

We fixed $\lambda_1 = 1$, $\lambda_2 = 0.15$, and $\beta_1 = 0.1$, and varied β_2 from 0.15 to 0.25 across a grid of values. For each value of β_2, we considered the same three count outcome distributions as in the main simulation study: Poisson (var = mean), over-dispersed Poisson (var $\propto$ mean), and modified negative binomial (var $\propto$ mean^2), each fitted using a log link. We then assessed the performance of 2SLiP and P2R2 by comparing the approximate sample sizes n_ϕ and n_R, as well as the resulting empirical power, against the true sample size n and its empirical power across varying values of β_2. Figure S.1 displays the simulation results under the log link.

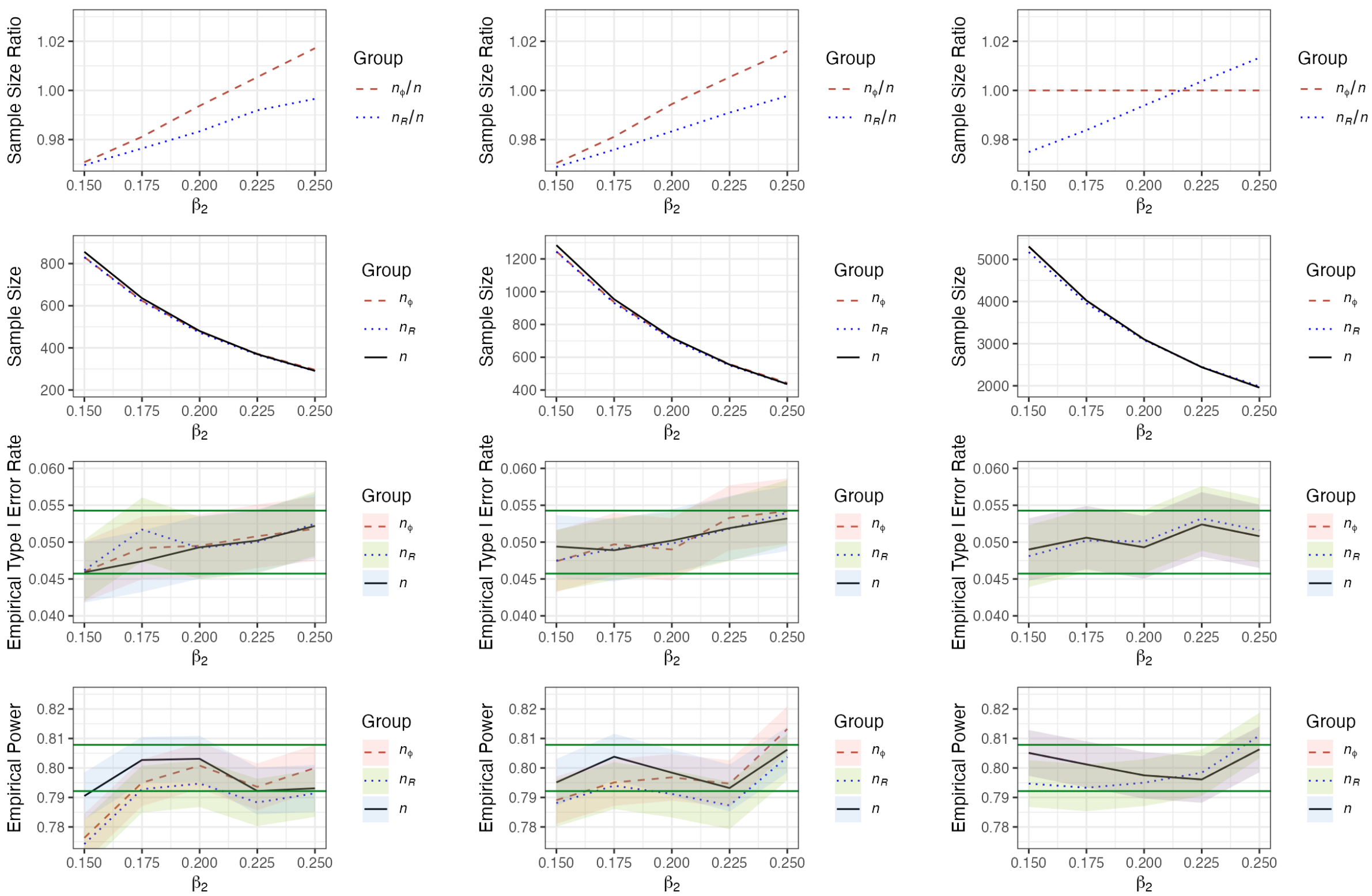

(a) var = mean (b) var $\propto$ mean (c) var $\propto$ mean^2

Figure S.1: The empirical type I error rates and power of the Wald test based on the simulated count data for three cases of outcome variables, using a log link and sample sizes n, n_ϕ, and n_R. The X-axis represents the coefficient β_2. The shaded envelopes represent the 95% confidence intervals of the empirical type I error rate and empirical power, respectively. The two horizontal lines in each panel indicate the 95% confidence interval around the corresponding target level (0.05 for type I error and 0.8 for power), serving as a benchmark for good calibration. Results falling within these horizontal lines indicate well-calibrated performance.

S.7.1.2 Identity Link

Under the identity link, we set $\lambda_1 = 4$, $\lambda_2 = 0.4$, and $\beta_1 = 0.4$, and allowed β_2 to range from 0.6 to 1.2. As with the log link, the same three count outcome distributions were examined. This setting provides a complementary view where the link function is linear, favoring the performance of the P2R2 approximation. We again compared n, n_ϕ, and n_R and their associated empirical powers across the range of β_2 values. Figure S.2 shows the corresponding results for the identity link.

S.7.2 Positive Continuous Outcomes

For positive continuous outcomes, we considered gamma distributions with variance proportional to the mean and to the squared mean, using the same log link function as in Section 3.1.2. Coefficients were set to $\lambda_1 = 1$, $\lambda_2 = 0.15$, and $\beta_1 = 0.1$, with β_2 ranging from 0.05 to 0.2. Figure S.3 presents the results for the continuous-outcome settings.

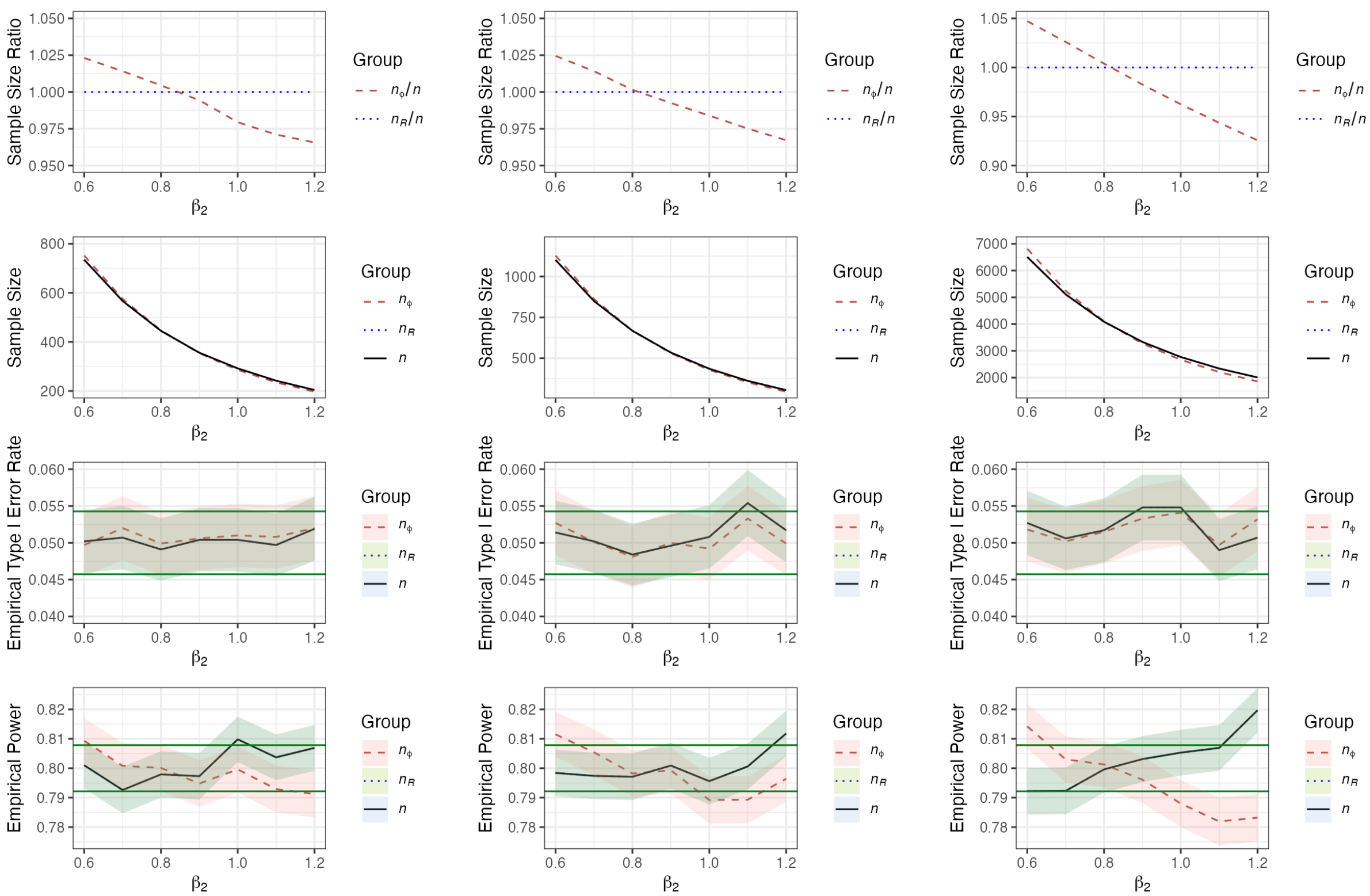

(a) var = mean (b) var $\propto$ mean (c) var $\propto$ mean^2

Figure S.2: The empirical type I error rates and power of the Wald test based on the simulated count data for three cases of outcome variables, using a identity link and sample sizes n, n_ϕ, and n_R. The X-axis represents the coefficient β_2. The shaded envelopes represent the 95% confidence intervals of the empirical type I error rate and empirical power, respectively. The two horizontal lines in each panel indicate the 95% confidence interval around the corresponding target level (0.05 for type I error and 0.8 for power), serving as a benchmark for good calibration. Results falling within these horizontal lines indicate well-calibrated performance.

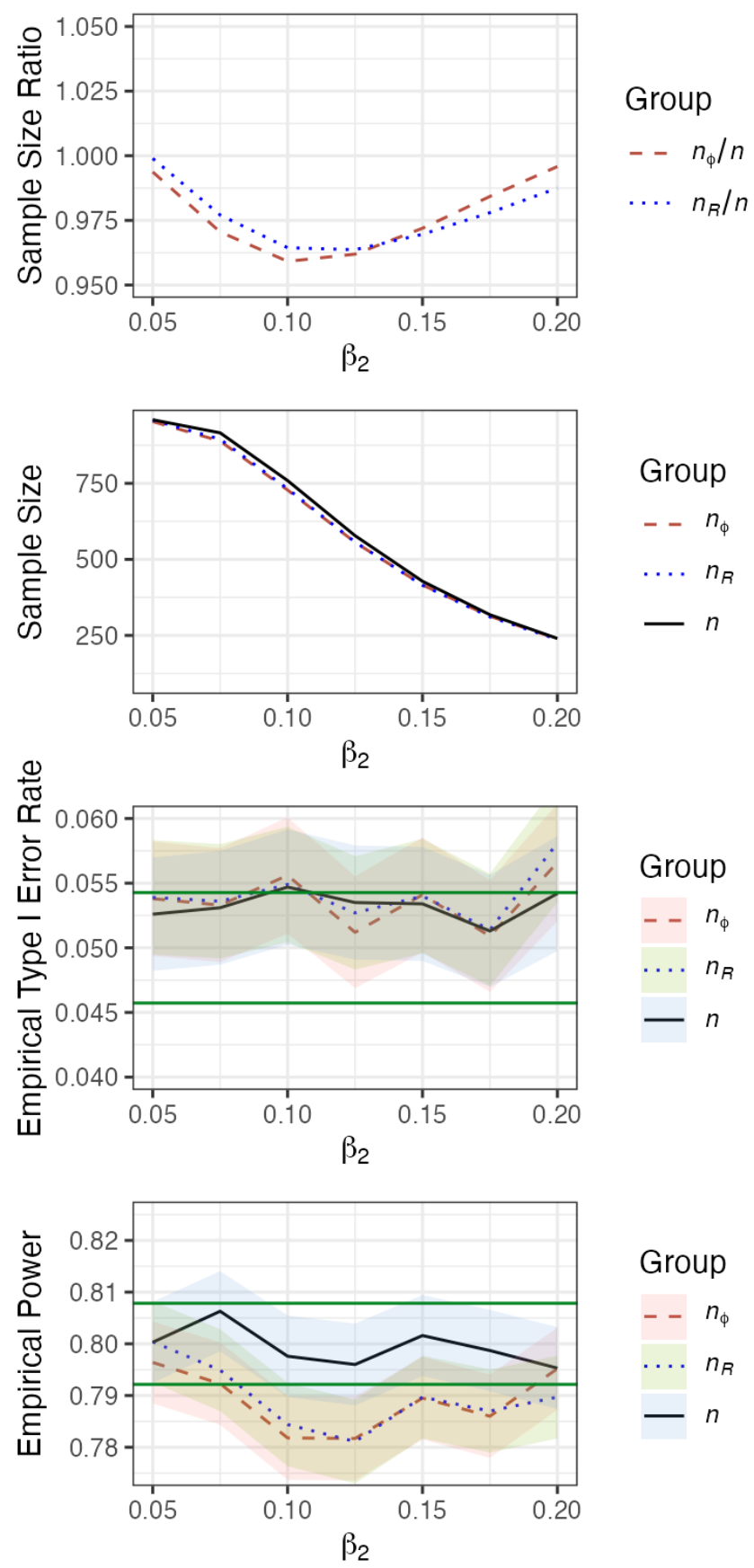

(a) var ∝ mean

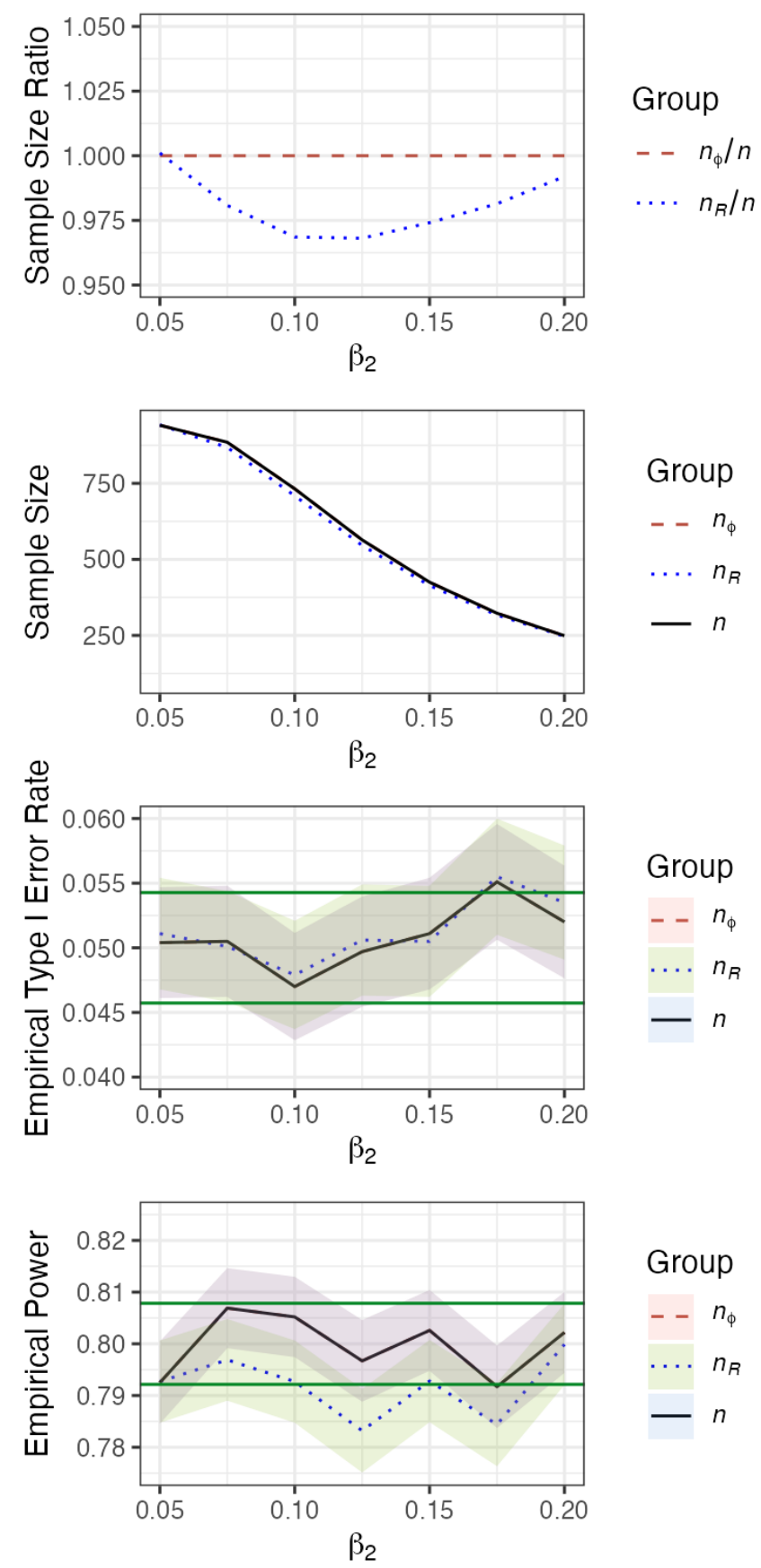

(b) var ∝ mean^2

Figure S.3: The empirical type I error rates and power of the Wald test based on the simulated positive continuous data for two cases of outcome variables, using a log link and sample sizes n, n_ϕ, and n_R. The X-axis represents the coefficient β_2. The shaded envelopes represent the 95% confidence intervals of the empirical type I error rate and empirical power, respectively. The two horizontal lines in each panel indicate the 95% confidence interval around the corresponding target level (0.05 for type I error and 0.8 for power), serving as a benchmark for good calibration. Results falling within these horizontal lines indicate well-calibrated performance.

References

McCullagh, P. (1983). Quasi-likelihood functions. *The Annals of Statistics*, pages 59–67.

Mccullagh, P. and Nelder, J. (1989). *Generalized linear models*. CRC press.
